# Molecular systematics of the *Sicista tianschanica* species complex: a contribution from historical DNA analysis

**DOI:** 10.7717/peerj.10759

**Published:** 2021-01-12

**Authors:** Vladimir S. Lebedev, Yulia Kovalskaya, Evgeniya N. Solovyeva, Elena D. Zemlemerova, Anna A. Bannikova, Mikhail Yu Rusin, Vera A. Matrosova

**Affiliations:** 1 Zoological Museum, Lomonosov Moscow State University, Moscow, Russia; 2 Severtsov Institute of Ecology and Evolution, Russian Academy of Sciences, Moscow, Russia; 3 Faculty of Biology, Lomonosov Moscow State University, Moscow, Russia; 4 Research and International Cooperation Department, Kiev Zoo, Kiev, Ukraine; 5 Schmalhausen Institute of Zoology, Kiev, Ukraine; 6 Engelhardt Institute of Molecular Biology, Russian Academy of Sciences, Moscow, Russia

**Keywords:** Cryptic species, Chromosomal variation, Natural history collections, Molecular diagnosis, Dipodoidea, Central Asia

## Abstract

The Tianshan birch mouse *Sicista tianschanica* is an endemic of the Central Asian mountains and has previously been shown to include several karyomorphs (“Terskey”, “Talgar”, “Dzungar”); however, the taxonomic status of these forms has remained uncertain. We examined the genetic variation in *S. tianschanica* based on historical DNA samples from museum collections, including the type series. Mitochondrial and nuclear data indicated that the species complex includes two major clades: Northern (N) and Southern (S) (*cytb* distance 13%). The N clade corresponds to the “Dzungar” karyomorph (Dzungar Alatau, Tarbagatay). The S clade is comprised of four lineages (S1–S4) divergent at 6–8%; the relationships among which are resolved incompletely. The S1 lineage is found in eastern Tianshan and corresponds to the nominal taxon. The S2 is distributed in central and northern Tianshan and corresponds to the “Terskey” karyomorph. The S3 is restricted to Trans-Ili Alatau and belongs to the “Talgar” karyomorph. The S4 is represented by a single specimen from southeastern Dzungar Alatau with "Talgar" karyotype. No interlineage gene flow was revealed. The validity of *S. zhetysuica* (equivalent to the N clade) is supported. Based on genetic and karyotypic evidence, lineages S2 and S3 are described as distinct species. The status of the S4 requires further investigation.

## Introduction

**Figure 1:**
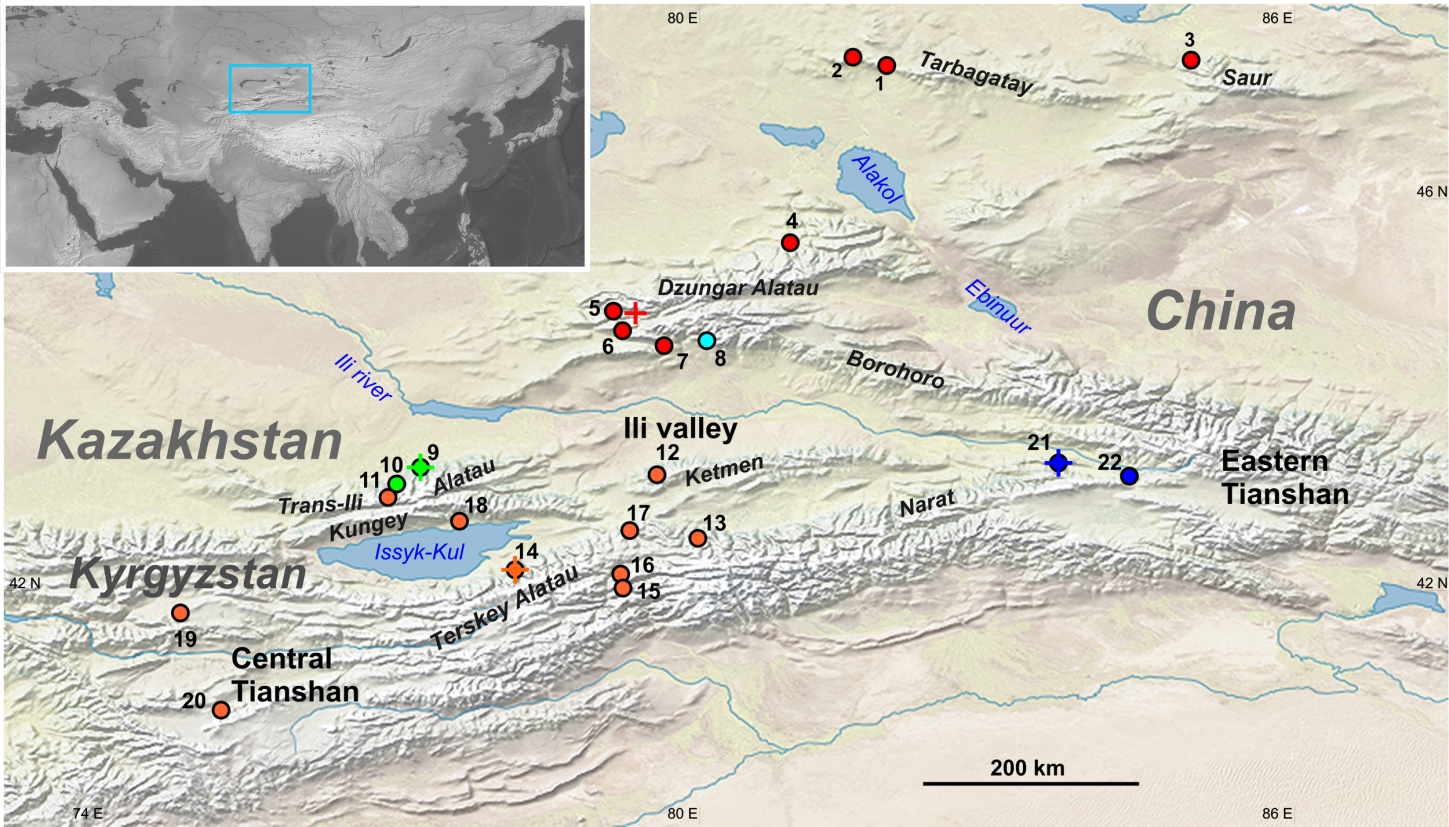
Geographic distribution of sampling localities and genetic lineages of the *Sicista tianschanica* sensu lato. Locality names are given in Table 1. Colours correspond to genetic lineages: red—N, blue—S1, orange—S2, green—S3, cyan—S4. Crosses designate type localities of *S. tianschanica* s.s. (loc. #21), *S. terskeica* sp.nov. (loc. #14), and *S talgarica* sp.nov. (loc. #9). Red cross indicates the type locality of *S. zhetysuica*.

The value of natural history collections as repositories of biodiversity information is constantly increasing. To a large extent, this is associated with their new role as a source of archive DNA for evolutionary and taxonomic research (Payne & Sorenson, 2002; Irestedt et al., 2006; Rowe et al., 2011; Bi et al., 2013). In particular, genetic data obtained from museum specimens are often indispensable for studies of rare, protected, or poorly accessible species (Ruane & Austin, 2017; Bannikova et al., 2019; Tsai et al., 2019; Turvey et al., 2019). Within the latter category, several groups of mammals are endemic to mountain regions of Central Asia (Tibet, the Himalayas, Tianshan, Altai), including several insufficiently studied species of birch mice (*Sicista* Griffith, 1827; Sminthidae; Rodentia). One of these is the Tianshan birch mouse *Sicista tianschanica* (Salensky, 1903), which is restricted to Tianshan, adjacent Dzungar Alatau and Tarbagatay (East Kazakhstan, West China, Kyrgyzstan; see Fig. 1), where it inhabits mountain grasslands and shrublands at the altitudes of 1,200–3,500 m a.s.l.

**Table 1:** Information on the material analyzed: ID of specimens, geographic origin, collection numbers, GenBank accession numbers.

ID, *karyotype*	Voucher	Locality, Lat/Long, Year Karyomorph (following Shenbrot et al., 1995)	*cytb*		*IRBP*		*BRCA1*	
			Acc. no.	True length	Acc. no.	Genotype, true length	Acc. no.	Genotype, true length
Trb_A1	ZMMU S-146639	Kazakhstan, Tarbagatai, East Kazakhstan reg., Urzhar dis., Kazymbet (Alexeyevka) **[1]** 47.30N 81.58E (1988) “Dzungar”	MT418257	1,140	MT418302	**N1/N1** 986 (24–653, 748–1,102)	MT418290	**N1/N1** 650
Trb_A2	ZMMU S-146647		MT418258	491 (255–745)				
Trb_U	ZMMU S-77367	Kazakhstan, Tarbagatai, East Kazakhstan reg., Urzhar dis., Alamdy **[2]** 47.37N 81.22E (1956)	MT418259	230 (255–484)				
Sau_Zh1	ZMMU S-146650	Kazakhstan, Saur, East Kazakhstan reg., Zaysan dis., Zhanturmys **[3]** 47.33N 85.00E (1988) “Dzungar”	MT418260	1,140	MT418303	**N1/N1** 1,047 (24–200, 211–905, 928–1,102)	MT418291	**N1/N1** 650
Sau_Zh2	ZMMU S-146651		MT418261	471 (255–461, 494–759)				
DzA_L	ZMMU S-96481	Kazakhstan, Dzungar Alatau, Almaty reg., Alakol dis., Lepsy (Lepsinsk) **[4]** 45.42N 80.67E (1967)	MT418262	204 (281–484)				
DzA_T1 *dzungar*	ZMMU S-202212	Kazakhstan, Dzungar Alatau, Almaty reg., Tekeli **[5]** 44.80N 79.00E (1987) “Dzungar”	MT418263	1,128 (1–483, 496–1,140)	MT418304	**N1/N2** 563 (1–200, 734–1,096)		
DzA_T2 *dzungar*	ZMMU S-143126		MT418264	477 (255–484, 495–741)				
DzA_A1	ZMMU S-146655	Kazakhstan, Dzungar Alatau, Almaty reg., Kerbulak dis., Aral-Tyube **[6]** 44.60N 79.17E (1988) “Dzungar”	MT418265	1,130 (1–484, 495–1,140)	MT418305	**N3/N3** 1,021 (24–652, 712–1,102 )	MT418292	**N3/N3** 650
DzA_A2	ZMMU S-146656		MT418266	490 (255–479, 495–759)				
DzA_P	ZMMU S-77366	Kazakhstan, Dzungar Alatau, Almaty reg., Panfilov dis., Ayaksaz **[7]** 44.48N 79.62E (1958)	MT418267	490 (255–480, 495–758)				
DzA_Zh talgar*	ZMMU S-202164	Kazakhstan, Dzungar Alatau, Almaty reg., Panfilov dis., Tyshkan riv. **[8]** 44.53N 80.10E (1988) “Talgar”	MT418268	1,067 (1–483, 557–1,140)	MT418306	**Z1/Z2** 591 (1–200, 712–1,102)	MT418293	**Z1/Z1** 195 (1–195)
TIA_T1 *talgar*	ZMMU S-202211	Kazakhstan, Trans-Ili Alatau, Almaty reg., Talgar dis., Almaty nat.res. **[9]** 43.27N 77.32E (1987) “Talgar”	MT418269	1,140	MT418307	**T2/T2** 591 (1–200, 712–1,102)	MT418294	**T1/T2** 481 (1–177, 347–650)
TIA_T2	ZMMU S-142913		MT418270	505 (255–759)				
TIA_T3 *talgar*	ZMMU S-142914		MT418271	1,140	MT418308	**T1/T1** 1,037 (24–653, 665–717, 749–1,102)	MT418295	**T1/T1** 650
TIA_T4	ZMMU S-142916		MT418272	1,140	MT418309	**T1/T1** 1,079 (24–1,102)	MT418296	**T1/T1** 650
TIA_A	ZMMU S-54768	Kazakhstan, Trans-Ili Alatau, Almaty reg., M Almaatinka riv., 15 km. S Almaata **[10]** 43.15N 77.07E (1952)	MT418273	212 (283–494)				
TIA_B	ZMMU S-64956	Kazakhstan, Trans-Ili Alatau, Almaty reg., Bol. Almaatinskoe lake **[11]** 43.05N 76.98E (1960)	MT418274	302 (275–576)				
Ktm_S	ZMMU S-148430	Kazakhstan, Ketmen, Almaty reg., Uygur dis., Syumbe **[12]** 43.25N 79.47E (1989) “Terskey”	MT418275	1,140	MT418310	**C1/C2** 1,075 (24–200, 205–1,102)	MT418297	**C1/C1** 641 (1–497, 507–650)
Ter_B1	ZMMU S-148433	Kazakhstan, Terskey Alatau, Almaty reg., Raiymbek (Narynkol) dis. Bayankol riv. **[13]** 42.58N 80.00E (1989) “Terskey”	MT418276	1,140	MT418311	**C1/C3** 1079 (24–1,102)	MT418298	**C1/C1** 650
Ter_B2	ZMMU S-148432		MT418277	505 (255–759)				
Ter_C *terskey*	ZMMU S-202206	Kyrgyzstan, Terskey Alatau, Issyk-Kul reg., Jeti-Oguz (Pokrovka) dis., Chon-Kyzyl-Su **[14]** 42.28N 78.12E (1977) “Terskey”	MT418278	1,140	MT418312	**C5/C5** 804 (1–413, 712–1,102)	MT418299	**C2/C2** 378 (1–378)
Ter_S1 *terskey*	ZMMU S-148428	Kyrgyzstan, Terskey Alatau - Saryzhaz, Issyk-Kul reg., Aksu dis., Saryzhaz bas., M. Taldysu riv. **[15]** 42.10N 79.13E (1989) “Terskey”	MT418279	423 (255–477, 535–734)				
Ter_S2	ZMMU S-78241	Kyrgyzstan, Terskey Alatau - Saryzhaz, Issyk-Kul reg., Aksu dis., Saryzhaz bas., Mukachi **[16]** 42.20N 79.12E (1962)	MT418280	421 (255–472, 528–730)				
Ter_U1 *terskey*	ZMMU S-148426	Kyrgyzstan, Terskey Alatau, Issyk-Kul reg., Aksu dis., Karkara bas., Uchkashka riv. **[17]** 42.65N 79.22E (1989) “Terskey”	MT418281	1,140	MT418313	**C2/C4** 1079 (24–1,102)	MT418300	**C1/C1** 650
Ter_U2	ZMMU S-148429		MT418282	480 (255–734)				
Kng_A	ZMMU S-136513	Kyrgyzstan, Kungey Alatau, Issyk-Kul reg., N bank Issyk-kul lake, Ananievo 20 km. **[18]** 42.78N 77.75E	MT418283	210 (275–484)				
CTS_K *terskey*	ZMMU S-148425	Kyrgyzstan, Central Tianshan, Naryn reg., Jumgal dis., Kara-Kiche **[19],** 41.75N 74.88E (1989) “Terskey”	MT418284	1,140	MT418314	**C1/C1** 1079 (24–1,102)	MT418301	**C1/C1** 638 (1–638)
CTS_A1	ZMMU S-58792	Kyrgyzstan, Central Tianshan, Naryn reg., At-Bashy dis., Akbeit can. **[20]** 40.83N 75.12E (1954)	MT418285	74 (87–160)				
CTS_A2	ZMMU S-58785		MT418286	74 (87–160)				
CTS_A3	ZMMU S-58788		MT418287	74 (87–160)				
ETS_T1	ZIN 2271	China, Eastern Tianshan, Xinjiang prov., Ili pref., Tzanma riv. **[21]** 43.33N 83.50E (1876)	MT418288	408 (87–160, 426–576, 678–759, 828–928)				
ETS_T2	ZIN 2273		MT418289	402 (87–160, 426–576, 678–753, 828–928)				
NT0609AQ10		China, Eastern Tianshan, Xinjiang prov., Ili pref., Nararti **[22]** 43.22N 84.32E	KM397204		KM397153	**E1/E1**	KM397290	**E1/E2**
NT0609BR03			KM397205		KM397154	**E1/E1**	KM397291	**E1/E1**
Trb_3		Kazakhstan, Tarbagatai, East Kazakhstan reg., Urzhar dis.	KY967410		KY967480	**N1/N1**	KY967462	**N1/N2**
St_RA		Sine loco	KY754150					
St_Sc		China, Xinjiang prov.			JF938868	Unphased	JF938765	**N4/N5**
DzA_DK		Kazakhstan, Dzungar Alatau			AF297288	**N4/N5**		
**Outgroups**								
MK0509BL02		*Sicista concolor*			KM397167		KM397294	
JJSA456			KJ648496					
Sc_07		*S. caudata*	MK259964				MK259972	
Sc_17					MK259969			
ZMMU S-136036		*S. napaea*, Altai, Shebalino 10 N						

The existing taxonomic arrangement within *Sicista* is, in general, based on karyotypes (Sokolov, Kovalskaya & Baskevich, 1987; Baskevich, 2016). Thus, among thirteen species of birch mice recognised in Mammal Species of the World (Holden & Musser, 2005), six species were originally diagnosed using karyotypic traits (Baskevich, 2016). Concerning the Tianshan birch mouse (*S. tianschanica*), its taxonomy seems to require revision, as chromosomal studies published nearly three decades ago (Sokolov & Kovalskaya, 1990) demonstrated that this species includes three well-differentiated karyomorphs, which were named “Terskey” (2*n* = 32, NFa = 56, 10M + 12SM + 4ST + 4A), “Dzungar” (2*n* = 34, NFa = 54, 10M + 10SM + 2ST + 10A) and “Talgar” (2*n* = 32, NFa = 56, 12M + 12SM + 2ST + 4A). The distribution of these chromosomal variants is allopatric. The “Terskey” karyomorph occupies central and northern Tianshan, the “Dzungar” is distributed in Dzungar Alatau, Tarbagatay and Saur, whereas the “Talgar” was found in two localities in Trans-Ili Alatau and southeastern Dzungar Alatau (Fig. 1). The three karyomorphs do not show complete arm homology and, hence, the differences in chromosome morphology cannot be explained by Robertsonian rearrangements only (for details see Fig. S1 and comment). Based on these results it was suggested that the Tianshan birch mouse may represent a species complex rather than a single species (Shenbrot et al., 1995). However, none of these forms have been described as a new species or subspecies, in part because the karyotype of the nominal form from eastern Tianshan (Xinjiang, China) remains unknown.

This problem could be approached from a molecular perspective; however, molecular data on Tianshan birch mice are scarce, being restricted to several specimens from few localities (four specimens from three localities at most). The multilocus analysis of the phylogenetic relationships in *Sicista* identified *S. tianschanica* as the sister group for all other members of this genus (Pisano et al., 2015; Lebedev et al., 2019). Furthermore, preliminary evidence for deep divergence within the Tianshan birch mouse clade is suggestive of cryptic speciation (Rusin et al., 2018; Cserkész et al., 2019; Lebedev et al., 2019). However, the correlation between molecular and chromosome variation, as well as the general pattern of genetic variation across the range are unknown. Sufficient de novo sampling would require a considerable trapping effort in hard-to-reach areas. Using an alternative approach, we addressed the two aforementioned tasks through an analysis of historical DNA from museum specimens, many of which belong to known karyomorphs. The taxonomy of the group will be discussed using genetic data obtained for the type specimens of *S. tianschanica* (Salensky, 1903).

## Materials and Methods

### Molecular samples

A total of 46 specimens stored in the Section of Mammalogy of the Zoological Museum of Moscow University (ZMMU) and Mammalian Collection of the Zoological Institute of Russian Academy of Sciences, Saint Petersburg (ZIN) were included in the molecular analyses. In most cases, small sections of skin (approximately 2 × 2 mm) from the rostrum or ventral region were sampled. Less often, if the skin was absent, bone samples (atlas or mandible and braincase fragments) were used. Most of the birch mice were collected in the 1950s–1980s, except for the holotype (lectotype ZIN 2271) and the two paratypes (paralectotype ZIN 2272, 2273) of *S. tianschanica* (Salensky, 1903), which were collected in 1876. Twenty-one specimens originated from chromosomally examined populations, and the karyotype was known for nine of those (Sokolov & Kovalskaya, 1990; Shenbrot et al., 1995). Detailed information on each specimen is provided in Table 1.

### DNA extraction, amplification and sequencing

We analysed the mitochondrial cytochrome b gene (*cytb*) and fragments of two nuclear genes: exon 1 of the interphotoreceptor binding protein gene (IRBP, partial exon 1) and exon 11 of the breast cancer type 1 susceptibility protein gene (BRCA1, partial exon 11). Nuclear genes were sequenced only for a subset of the sample due to DNA quality issues. DNA was extracted and purified using the QIAamp DNA MiniKit (Qiagen, Germany), including an overnight lysis step at 56 °C and longer incubation with EB buffer (5 min) during the purification step. The DNA was highly degraded; thus, only short fragments (~100–300 bp) were obtained using a combination of 17 pairs of internal primers designed for this study (Table S1).

The PCR program for amplification of short fragments included an initial denaturation at 95 °C for 3 min, 45 cycles at 95 °C for 30 s, at the annealing temperature (Table S2) for 30 s, at 72 °C for 30 s, and a final extension at 72 °C for 6 min. All stages of the extraction and PCR processes included a negative control run in parallel. To avoid contamination, extraction, and amplification of the DNA from the museum specimens were conducted in the ZMMU Laboratory of Historical DNA, equipped exclusively for procedures with museum DNA specimens, where no previous analysis on fresh tissues had been performed. PCR products were separated in 1.5% agarose gel stained with ethidium bromide, visualized in UV light, cut off, and purified using a GeneJET Gel Extraction Kit (Thermo Fisher Scientific, Waltham, MA, USA), according to the manufacturer’s instructions. The nucleotide sequences were determined using an ABI PRISM 3,500×L automatic sequencer with BigDye Terminator Chemistry v. 3.1 (Applied Biosystems, Foster City, CA, USA) using PCR primers (Table S1).

All sequences were assembled in SeqMan (DNASTAR, Lasergene, Madison, WI, USA) and the alignments were built in BioEdit v. 7.0.4.1 (Hall, 1999). The sequences obtained in this study were deposited in GenBank (accession numbers: MT418257−MT418314, Table 1). Additional data retrieved from GenBank included six sequences of *cytb*, six sequences of BRCA1 and seven sequences of IRBP (Table 1). *S. caudata*, *S. napaea* and *S. concolor* were used as outgroups.

### Phylogenetic analyses of mitochondrial data

Phylogenetic trees were inferred under maximum parsimony (MP), maximum likelihood (ML) and Bayesian inference (BI) criteria. MP reconstructions were conducted in PAUP* 4.0b10 (Swofford, 2003) using the following options: random addition sequence with 1,000 replicates, branch-swapping with tree bisection-reconnection, “Multrees” option not invoked. The analysis yielded multiple equally parsimonious topologies from which a 50% majority rule consensus tree was reconstructed. To assess clade stability, 1,000 bootstrap pseudoreplicates were analysed.

Maximum likelihood reconstructions were performed in IQTree version 1.6 (Nguyen et al., 2015). The ModelFinder routine (Kalyaanamoorthy et al., 2017) was used to identify the optimum partitioning scheme and best-fit substitution models for each subset under the Bayesian information criterion (see Table S3 for details). Clade stability was estimated using Ultrafast Bootstrap (Minh, Nguyen & Von Haeseler, 2013) with 10,000 replicates.

Bayesian tree reconstruction was conducted in MrBayes 3.2.7 (Ronquist et al., 2012) assuming separate models for each of the codon positions. The model choice was based on the results of ModelFinder. Compound Dirichlet priors for branch lengths combined with a gamma prior to tree length were employed. All parameters, except branch lengths, were unlinked across subsets. The analysis included two independent runs of four chains with the default heating scheme. Chain length was set to 5,000,000 generations, sampling at every 2,000 generations. Tracer 1.7 (Rambaut & Drummond, 2005) was used to check for convergence and determine the necessary burn-in fraction, which was 10% of the chain length. Uncorrected genetic p-distances among haplotypes were calculated using PAUP* 4.0b10.

To estimate the divergence times among major genetic lineages a chronogram was generated in BEAST version 1.10 (Drummond et al., 2012) under a strict clock. The strict clock assumption was tested in PAML v. 4.9 (Yang, 2007) with the use of hierarchical likelihood ratio tests (hLRT). The tree was calibrated based on the estimated age of the split between the two main clades of *S. tianschanica* (~3.0 Mya; 95% HPD: 2.3–3.9). The latter estimate was obtained in a previous multilocus study (Lebedev et al., 2019), sequences from which were included in our alignment. Considering the high saturation rate and relatively old age of the root, transitions at the third codon positions were excluded from the analysis. Only sequences longer than 1,000 bp were included, thus reducing the sampling to 19 specimens. Substitution models for the first and second codon positions were set in the ML analysis, rate variation for the third position transversions was assumed to follow a gamma distribution. The calibrated Yule tree prior was employed. Chain and burn-in lengths were set at 10,000,000 and 1,000,000 generations, respectively. Convergence diagnostics were performed using Tracer 1.7.

### Nuclear data analyses

In most sequences, no more than one heterozygous position was observed; hence, the allelic phase could be reconstructed unambiguously. In other cases (one BRCA1 and two IRBP sequences), phases were assigned arbitrarily, which could not appreciably affect the results. Median networks were reconstructed using TCS under the default options (Clement, Posada & Crandall, 2000) and visualized using tcsBU (Múrias dos Santos et al., 2016). Additionally, the ML tree was generated in IQTree based on the concatenated unphased sequences of BRCA1 and IRBP.

### Species delimitation and species tree reconstruction

To objectively identify boundaries between genetic lineages (i.e., potential species) we employed two species delimitation methods. First, we used the single-locus multi-rate Poisson Tree Process method (Kapli et al., 2017) as implemented in the online version of mPTP program (https://mptp.h-its.org/#/tree). Compared to other single locus tree-based methods, mPTP is less prone to over-split taxa (Kapli et al., 2017). The analysis was performed based on phylogenetic trees reconstructed from the *cytb* dataset. Both the MP majority rule consensus (with branch lengths estimated via Minimum Evolution algorithm) and the ML tree were used as input.

Next, we conducted species delimitation using a coalescent model-based multilocus approach implemented in the program BPP version 4.3 (Yang & Rannala, 2014; Flouri et al., 2018). The analyses were run both using fixed guide tree (A10) and unguided (A11). The A11 analysis performs joint species tree reconstruction and species delimitation. The MP majority rule consensus tree was used as the guide tree for the A10 analysis. The input data included three alignments (*cytb*, IRBP, BRCA1), nuclear sequences were phased as described above. Heredity scalars of 0.5 and 2.0 were used for the *cytb* and nuclear data, respectively. At the initial stage, each specimen was designated as an independent lineage. Two specimens of *S. tianschanica* for which *cytb* data were unavailable and all outgroups were excluded from the analyses.

Taking into account potential sensitivity to prior setting, we used diffuse priors for the parameters of the multispecies coalescent model. The population size parameters (*theta*s) were assigned the inverse-gamma prior IG (3, 0.01), with mean 0.005. To improve mixing, *theta* parameters were integrated analytically. The divergence time at the root of the species tree (*tau*0) was assigned the inverse-gamma prior IG (3, 0.2), with mean 0.1, while the other divergence time parameters were specified by the uniform Dirichlet distribution (Yang & Rannala, 2010). The mean rate across loci (*mu*) was fixed at 1. To accommodate for difference in substitution rate between mitochondrial and nuclear genes, rate variation across loci was modeled using gamma distribution with *alpha* parameter 0.5 (mean 1.0; variance 2.0). We used the uniform rooted tree prior. Sequence evolution was assumed to follow a strict clock model. GTR+G substitution model was applied. MCMC chain was set to 20 million and 2 million generations for A11 and A10, respectively, with burnin = 5% and number of samples = 2000. Each analysis was run twice using the reversible-jump Markov chain Monte Carlo (rjMCMC) algorithm 1 and once using rjMCMC algorithm 0. Convergence was assessed by comparing the consistency of posterior distributions. To estimate the species tree with posterior probabilities for clades, we constructed the majority rule consensus tree of the posterior sample of species trees generated by the A11 algorithm.

### Cranial variation

Cranial variation was assessed using 25 cranial measurements obtained from 50 specimens (ZMMU collection), including birch mice of all three known karyomorphs (Table S4). Genetic data were available for 13 specimens. Considering that the braincase and the zygomatic arch are fragile and often deformed in birch mice, many of the standard measurements were omitted, and additional dental measurements were included. The detailed information on the measurements is provided in Supplemental Material S3. The sample from Trans-Ili Alatau collected by Ognev was attributed to the “Talgar” karyomorph based on the results of preliminary discriminant analysis (results not shown).

The skulls of the holotype (ZIN 2271) and a paratype (ZIN 2273) of *S. tianschanica* (Salensky, 1903) were damaged and, hence, could not be included in the multidimensional morphometric analysis. Several dental measurements of the type specimens were used for comparison with other samples.

All dental and cranial measurements were obtained using the eyepiece reticle of the stereomicroscope (MBS-10) calibrated with digital callipers at several magnifications (×4.8, ×16, ×32 and ×56). The maximum resolution was ~1/70 mm.

The analyses were performed based on log-transformed values using routines implemented in STATISTICA v.8.0 (StatSoft, Tulsa, OK, USA). A principal component analysis (PCA) was used to obtain principal axes that summarized the directions of greatest variation within the total sample. The significance of differentiation among karyomorphs was tested using Hotelling’s test. To identify variables contributing to between-group separation, pairwise Mann–Whitney *U*-tests with Bonferroni corrections were performed. To identify size-independent differences, the general linear model (GLM) procedure was employed using karyomorph as the categorical predictor and condylo-incisive length (a proxy for size) as a continuous predictor. A preliminary GLM analysis using sex and karyomorph as predictors demonstrated a lack of cranial sexual dimorphism.

### Nomenclatural information

The electronic version of this article in Portable Document Format (PDF) will represent a published work according to the International Commission on Zoological Nomenclature (ICZN), and hence the new names contained in the electronic version are effectively published under that Code from the electronic edition alone. This published work and the nomenclatural acts it contains have been registered in ZooBank, the online registration system for the ICZN. The ZooBank LSIDs (Life Science Identifiers) can be resolved and the associated information viewed through any standard web browser by appending the LSID to the prefix http://zoobank.org/. The LSID for this publication is: urn:lsid:zoobank.org:pub:6F34EAF8-92B3-42BC-B07E-E6C16731A9A1. The online version of this work is archived and available from the following digital repositories: PeerJ, PubMed Central and CLOCKSS.

## Results

### Characteristics of *cytb* sequences

*Cytb* sequences were obtained for 32 specimens of *S. tianschanica* sensu lato, whereas for 11 specimens all attempts to amplify even smaller fragments were unsuccessful. In particular, no amplifiable DNA was yielded by the three specimens stored in 70% ethanol (ZMMU collection). Complete or near-complete *cytb* sequences (1,065–1,140 bp) were obtained for 13 specimens. For 14 specimens, we sequenced 1–3 fragments, ~200 bp each.

Only four small fragments (70–150 bp) could be amplified for the holotype (ZIN 2271) and one of the paratypes (ZIN 2273), no reliable data was recovered for the second paratype (ZIN 2272). The total length of the sequenced portion of the *cytb* is 408 bp for ZIN 2271 and 402 bp for ZIN 2273. Compared to all other birch mice analysed in our laboratory, all fragments from the type specimens are unique; therefore, contamination can be ruled out. The sequences contain no clear double C/T peaks, suggesting that the occurrence of sequencing artefacts because of cytosine deamination was unlikely.

Amplification was unsuccessful for five of the eight examined birch mice from Akbeit Canyon (loc. #20), whereas a single short fragment (74 bp) was obtained for the other three. The latter sequences are excluded from tree reconstructions; however, the data are sufficient for the identification of the lineage. The final alignment includes 31 sequences of *S. tianschanica* sensu lato from 21 localities representing all three known karyomorphs (Fig. 1).

### *Cytb* phylogenetic hypothesis and divergence times

**Figure 2:**
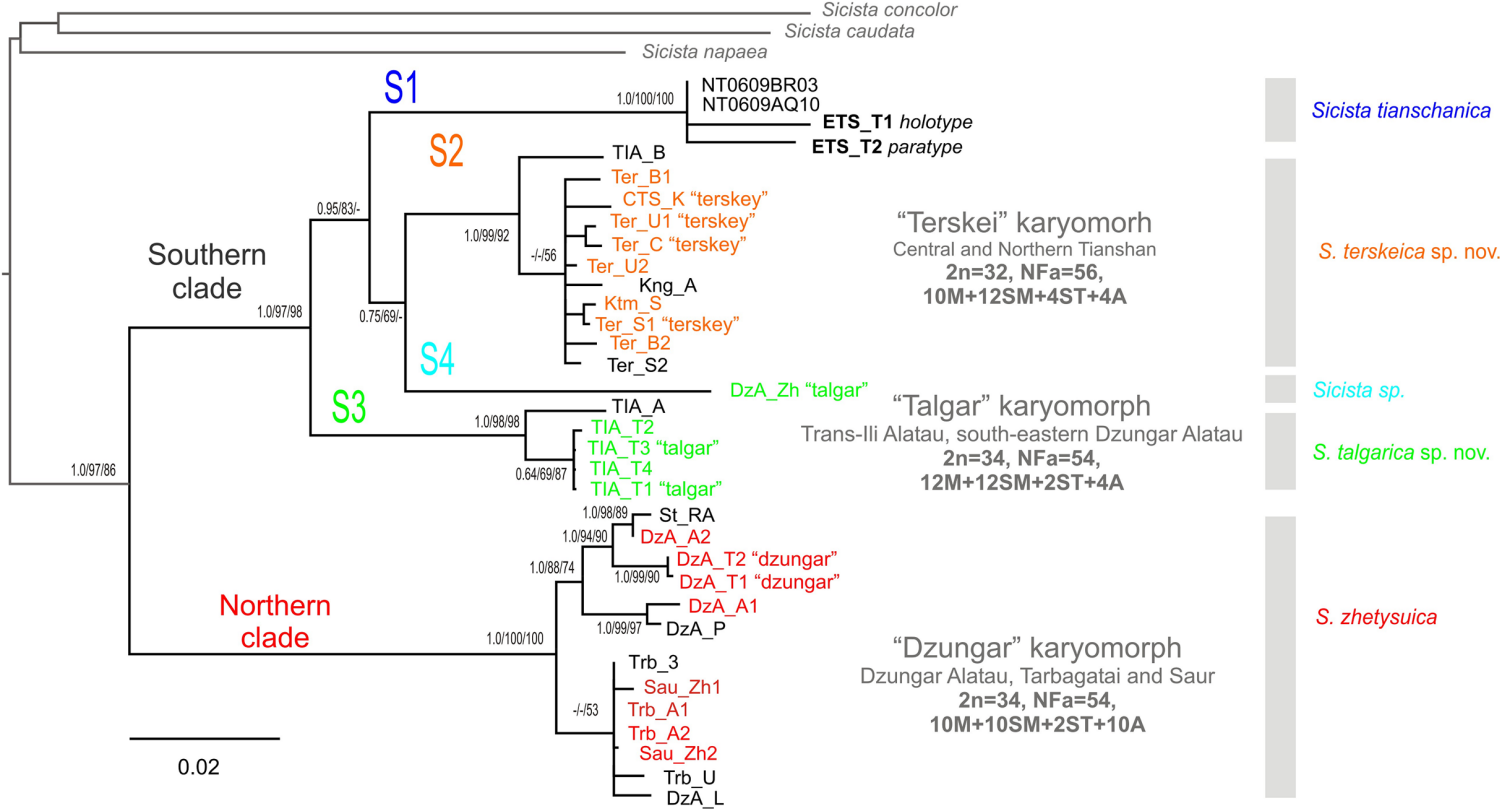
The Maximum Parsimony phylogeny of the *Sicista tianschanica* species group as inferred from the *cytb* data. Blanch lengths were estimated using Minimum Evolution algorithm in PAUP* 4.0. Numbers above/below branches are Bayesian posterior probabilities, and MP and ML bootstrap support values (>50%) (BI/ML/MP). Colours correspond to karyomorphs: red—“Dzungar”, orange—“Terskey”, green—“Talgar”. Specimen designations are as in Table 1 and include karyotype name for all chromosomally examined individuals. Genetic lineages are indicated above branches.

The phylogenetic trees inferred using the ML, MP and BI methods are highly similar and contain no strongly supported incongruent clades between the analyses. Within *S. tianschanica* sensu lato, all analyses (Fig. 2) recover two highly supported clades, which we designate Northern (N) and Southern (S), with 13.4% p-distance between them. The latter clade is divided into four well-supported lineages (S1–S4) divergent at 6.7–8.6%. In all three reconstructions, the S3 lineage is consistently placed as a sister to the other subclades (with support in ML and BI analyses); whereas the S4 and the S2 tend to form a cluster to the exclusion of the S1; however, the support for the latter relationship is low.

The N clade has a discontinuous distribution with one area in Dzungar Alatau and another in Tarbagatay and Saur (Fig. 1). In the MP tree, the N clade is subdivided into two haplogroups, which correspond to the two parts of the range except for the position of one specimen from northern Dzungar Alatau. The distance between the haplogroups is relatively large (2.2%); however, the monophyly of the Tarbagatay - Saur haplogroup has no bootstrap support. Moreover, in the ML and Bayesian trees, the latter group appears paraphyletic relative to the Dzungar Alatau haplogroup.

The S1 lineage, including the type specimens of *S. tianschanica*, is found in eastern Tianshan only. The S2 lineage is distributed more widely, occurring in several mountain ranges in central and northern Tianshan, including Terskey Alatau, Kungey Alatau, Ketmen and a single point in Trans-Ili Alatau (Fig. 1). The S3 lineage is restricted to Trans-Ili Alatau (two sites near the Talgar Mountains). The S4 lineage is represented by a single specimen collected in southeastern Dzungar Alatau (the Tyshkan River valley). Although members of different lineages can be found in the same mountain range (e.g., N and S4 in Dzungar Alatau, and S2 and S3 in Trans-Ili Alatau) no cases of sympatry are revealed.

The hLRT test indicates no significant departure from the strict clock model (*P* > 0.17).

**Figure 3:**
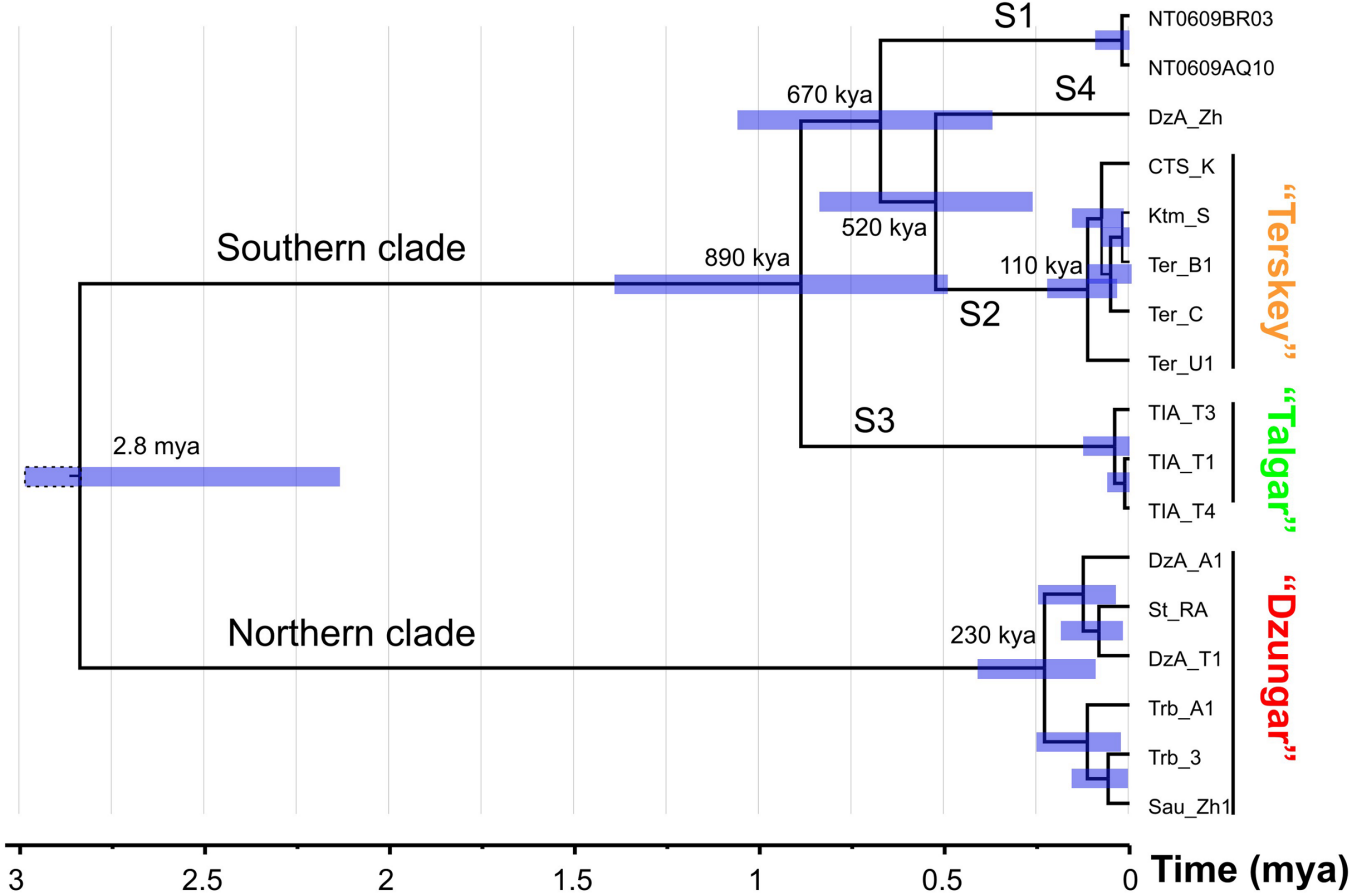
The chronogram (BEAST) of the divergence events in the *Sicista tianschanica* species group based on *cytb* data. Node bars correspond to 95% highest probability density intervals (HPD). The scale at the bottom indicates evolutionary time in million years.

The results of the divergence time analysis (Fig. 3) indicate that the splits among the S1–S4 lineages date back to the early Middle Pleistocene (520–890 kya). The estimated time of the most recent common ancestor (tmrca) of the N clade corresponds to the late Middle Pleistocene (ca. 230 kya). The tmrca of the S2 lineage is estimated at ca. 110 kya (early Late Pleistocene).

### Characteristics of nuclear sequences and nuclear phylogenetic hypothesis

IRBP sequences were obtained for 13 specimens of *S. tianschanica* sensu lato representing all known karyomorphs (Table 1). Total lengths of sequenced fragments vary from 563 to 1,079 bp. Thirty-six positions are variable and sequences of five specimens in this study are heterozygous.

BRCA1 was sequenced for 12 specimens. The total length of BRCA1 sequences is 195–650 bp. Twenty-two positions are variable and one newly examined specimen is heterozygous. For the type specimens, no sequences for IRBP and BRCA1 were obtained.

**Figure 4:**
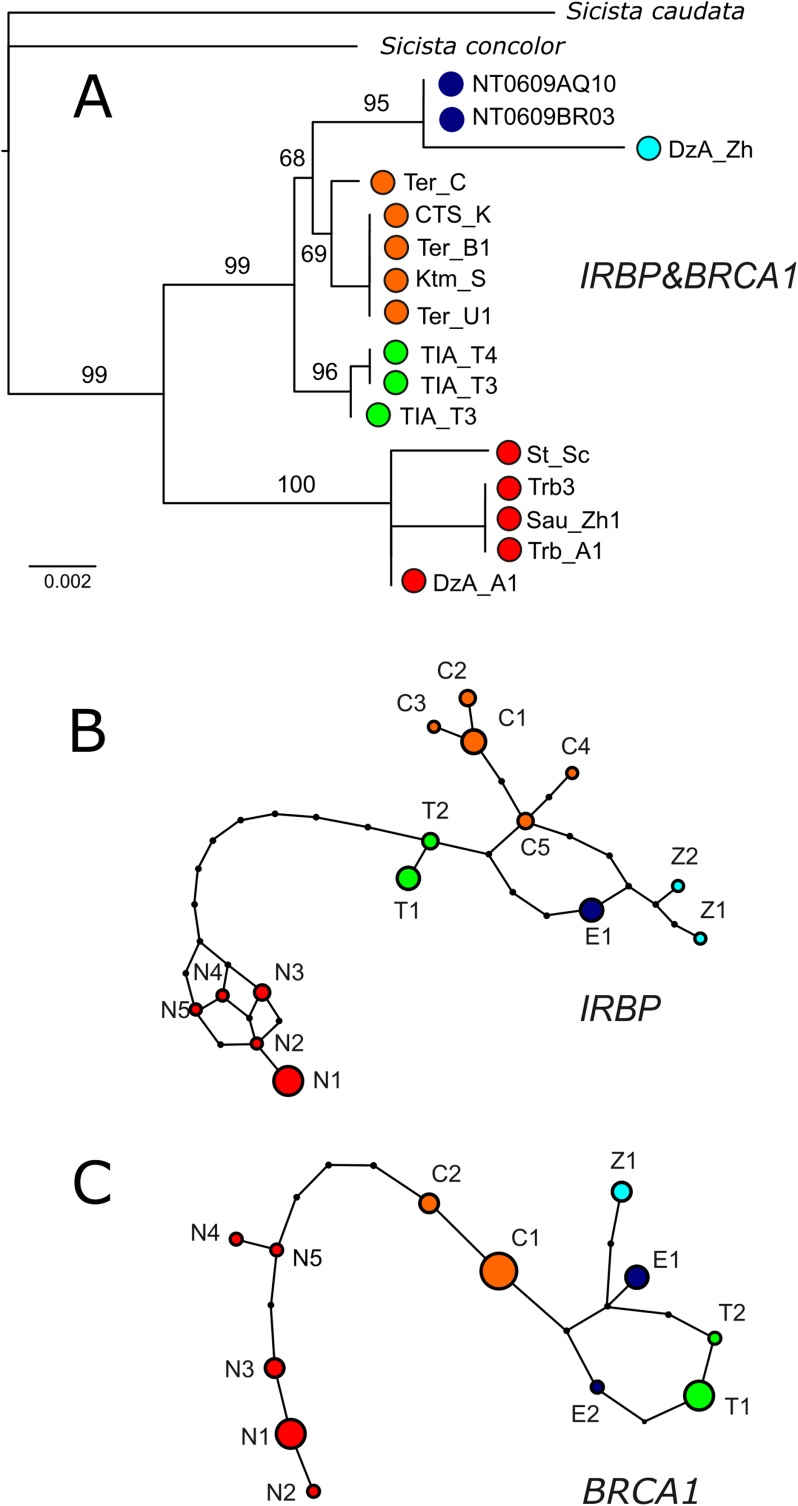
Results of phylogenetic analyses of nuclear data. (A) The ML tree reconstructed in IQTree from the concatenated alignment of *BRCA1* and *IRBP*. Ultrafast bootstrap support values are given above branches. Specimen designations are as in Table 1 and Fig. 2. Colours correspond to those in Fig. 1. (B & C) Haplotype networks depicting the relationships among alleles of nuclear genes as inferred by TCS. Allele designations are as in Table 1.

In both IRBP and BRCA1 networks and phylogenetic trees (Fig. 4), unique sets of alleles corresponding to each of the mitochondrial groups (N, S1, S2, S3, S4) are observed. No heterozygotes containing alleles specific to different lineages are revealed. The nuclear ML tree strongly supports the dichotomy between the N and S clades. All southern lineages are recovered as monophyletic, however, the relationships among them remain unresolved.

### Species delimitation and species tree analysis

The mPTP analyses with both ML and MP trees inferred five distinct lineages of *S. tianschanica* sensu lato corresponding to N, S1, S2, S3 and S4 (Fig. 2).

**Figure 5:**
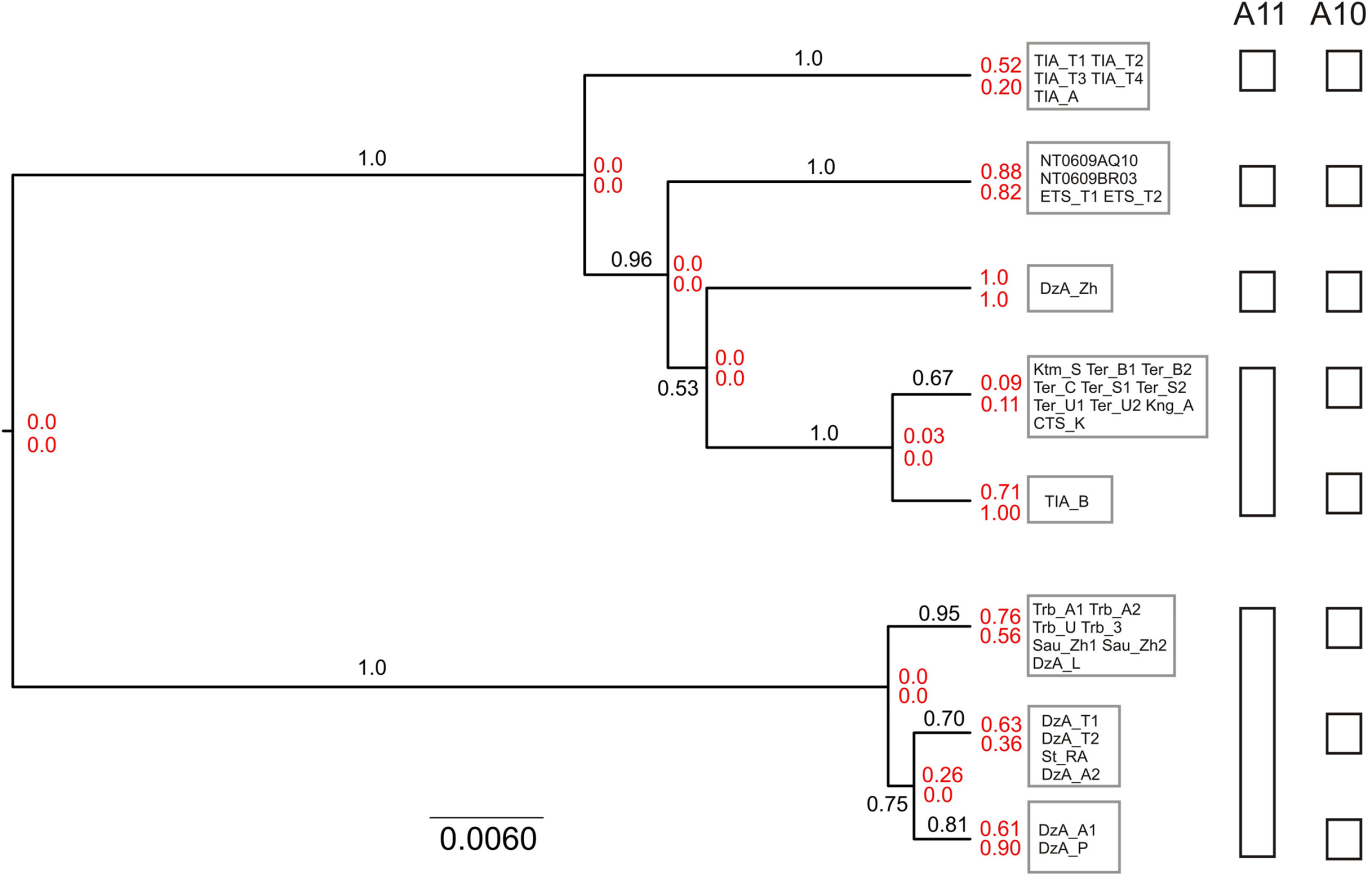
Results of species delimitation and species tree reconstruction in BPP. The tree was reconstructed using A11 method (simultaneous species delimitation and species tree inference). Numbers above or below branches indicate posterior probabilities for clades. Numbers at nodes (shown in red) correspond to posterior probabilities of node absence (i.e., probability of delimitation in which the clade downstream of node is a separate unsplit species; above—A11, below—A10). Details of delimitation within supported groups are not shown.

The results of unguided species delimitation in BP&P (A11 method) were largely consistent across runs. Each analysis yielded multiple delimitation schemes containing 6–18 putative lineages (mean 9.8). Posterior probabilities for all delimitation hypotheses were low (PP < 0.05). Nevertheless, the analyses indicated robust support (PP > 0.99) for the distinction of five main lineages (i.e., N, S1, S2, S3 and S4) (Fig. 5). Although in a large fraction of the posterior sample, these lineages (except S4) were split into several subgroups, this fine-scale delimitation is not fully significant due to low posterior probability for presence of nodes and insufficient support for clades in the species tree (e.g., subdivisions within the N and S2 lineages). Thus, the hypothesis that there are fewer than five potential species is rejected while the hypothesis that there are more than five species is neither rejected nor strongly supported.

The results of species delimitation using a fixed guide tree (A10 method) corroborated the separation of the five main lineages. At the same time, this method supported (PP > 0.95) additional putative species including three groups within the N clade and two groups within the S2 lineage. This result highlights the impact of topological uncertainty on species delimitation. The interpretation of the observed pattern is further complicated by the fact that BPP is known to oversplit species under a variety of conditions (Leaché et al., 2019).

The species tree reconstructed with BPP (A11 analysis) was congruent with the *cytb* tree, including that the S3 lineage was supported as the sister group to other southern lineages (Fig. 5). The relationships among the S1, S2 and S4 were not resolved with high support.

### Cranial variation

**Figure 6:**
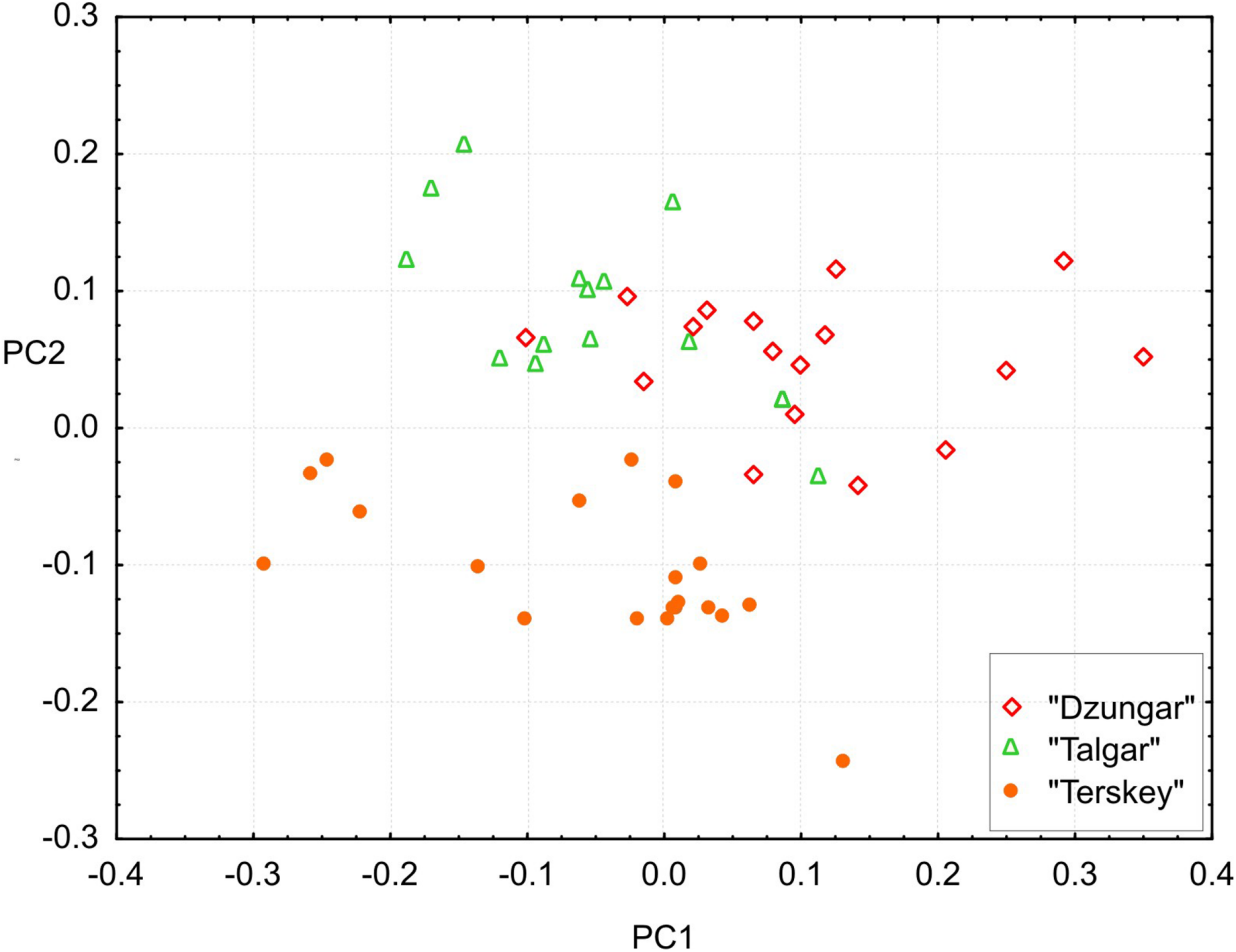
Projection of specimens onto the first and second principal components extracted from the matrix of covariances among 25 cranial measurements. Different symbols denote different karyomorphs (based on the specimen’s geographic origin).

All measurements, including toothrow lengths, overlap among the groups. However, the multivariate tests show that all three groups are significantly different (Hotelling’s test, *P* < 0.00001 for all pairwise comparisons).The results of the PCA (Fig. 6) demonstrate that the “Terskey” group is separate from the other two based on the combination of the first two principal components with the main contribution coming from PC2 (21.7% of total variance). No hiatus between “Talgar” and “Dzungar” is evident. Additional axes (not shown) do not improve discrimination between the groups. The first PC (36.3% of total variance) is negatively correlated with all measurements; thus, it reflects size variation.

The univariate results are summarized in Table S4. “Terskey” is distinguished by a wider palatine bridge (i.e., more widely separated molar tooth rows), a slightly narrower interorbital constriction, and a larger size of M3. The “Talgar” differs from the others by relatively wider nasals, whereas “Dzungar” is characterized by a more well-developed P4, as manifested by a significantly higher P4M3L/M1M3L ratio. The GLM results indicate significant effects of group for twelve variables. These results should be interpreted cautiously because variation among populations within chromosomal groups remains unstudied due to the small size of local samples; hence, the relative magnitudes of the intra- and intergroup variance components remain unknown.

Concerning the type series, in two of the three specimens (ZIN 2271 and 2273), the upper tooth row is notably longer (P4M3 = 3.73 and 3.78 mm, respectively) compared with that of all other Tianshan birch mice (range = 2.92–3.32). However, the diagnostic value of this difference is unclear because, in the other paratype (ZIN 2272), the tooth row is smaller (P4M3 = 3.21); genetic data for this specimen are lacking.

In addition, it should be noted that no distinct qualitative cranial traits (such as details of molar cusp morphology, cranial suture shape, etc.) that could be used to discriminate among the three studied chromosomal groups were revealed.

## Discussion

During the last two decades, mammal taxonomy is experiencing a dramatic acceleration in the rate of species description (Burgin et al., 2018). Nearly 250 new rodent species have been described during the last 20 years (D’Elía, Fabre & Lessa, 2019). Cryptic species that are detected due to the application of molecular and cytogenetic methods represent a substantial fraction of the newly discovered diversity. According to D’Elía, Fabre & Lessa (2019), ~70% and ~40% of recently described species were discovered with the use of molecular and karyotypic data, respectively.

It should be highlighted that the use of historical samples has been crucial in recent studies to reveal cryptic diversity within many branches of the rodent Tree of Life (De Abreu et al., 2020; Castañeda-Rico et al., 2020; Emmons & Fabre, 2018).

The genus *Sicista* is an illustrative example of a taxon with a high level of cryptic diversity. The genus comprises several species complexes (species groups), each consisting of 2–5 parapatric or allopatric, morphologically cryptic species (e.g., *S. betulina* complex, *S. subtilis* complex, *S. caucasica* complex). Within these groups, new species were originally detected using karyotypic evidence (Sokolov, Baskevich & Kovalskaya, 1981; Sokolov & Baskevich, 1988; Sokolov, Kovalskaya & Baskevich, 1989; Kovalskaya et al., 2011); subsequently, their validity was tested based on molecular data (Rusin et al., 2018; Lebedev et al., 2020), which, at the same time, allowed to identify new species (Cserkész, Rusin & Sramkó, 2016). There are good reasons to expect that, similarly to other groups of *Sicista*, the *S. tianschanica* species complex may harbor yet undescribed, cryptic species. In the next sections, we will analyze correspondence between molecular and chromosomal evidence available for *S. tianschanic*a sensu lato and, then, evaluate these data from a taxonomic perspective.

### Comparison between karyomorphs and genetic lineages

The results of the molecular analyses were generally consistent with predictions from chromosomal data, as illustrated by the lack of karyotype variation within the five robustly supported genetic lineages. The N clade fully corresponded to the “Dzungar” karyomorph (most of Dzungar Alatau, Tarbagatay, Saur), whereas the S2 lineage was equivalent to the “Terskey” karyomorph (Terskey Alatau, Kungey Alatau, central Tianshan, Ketmen). The karyotype of the nominal form (S1 lineage) remains unknown.

In contrast, the “Talgar” karyotype variant was shared by the S3 (Trans-Ili Alatau) and S4 (SE Dzungar Alatau) lineages, which are not the closest sister groups. The apparent paraphyly of the “Talgar” karyomorph can be explained by either assuming that the “Talgar” karyotype is plesiomorphic relative to the “Terskey” karyotype or by superficial parallelism of chromosome morphology (i.e., without true homology of all elements), which could potentially be revealed in the future with the use of differential staining or other techniques. Heretofore, it was difficult to interpret the pattern of distribution of the “Talgar” karyomorph because populations from Trans-Ili Alatau and Dzungar Alatau were separated by the wide (>80 km) valley of the Ili, which likely acts as an effective barrier for birch mice dispersal because of the prevalence of arid habitats. This problem is now resolved by the molecular data, which demonstrates that the animals with the “Talgar” karyotype distributed to the north and to the south of the Ili valley, in fact, represent two unrelated genetic lineages. At the same time, it has become clear that, in some lineages, the distribution range could be restricted to a relatively small area, as is the case for S3, which has currently only been found in the Talgar Mountains in Trans-Ili Alatau. An unexpected result was that the birch mouse collected in a chromosomally unstudied site (loc. #11) located just 30 km eastward from Talgar belonged to the S2 lineage. Thus, the distribution of all lineages, and especially of the least represented S1, S3 and S4 lineages, requires more detailed research.

From a phylogeographic perspective, the pattern of variation and distribution of the N clade deserves special note since this clade is found in both Tarbagatay and Dzungar Alatau mountains, separated by the Djungarian gate (Ebinur and Alakol depression) that is currently excessively arid for Tianshan birch mice. Occurrence of similar haplotypes in Tarbagatay and northern Dzungar Alatau suggests that these ranges were recently (presumably in Late Pleistocene) connected through a corridor suitable for dispersal of mesic small mammals. This evidence contributes to our knowledge of the phylogeography of the Tianshan region, which is still grossly understudied. The available phylogeographic data on terrestrial vertebrates are fragmentary (Zhou, Turdy & Halik, 2015; Orlova et al., 2017; An et al., 2020) and do not include any cases for small mammals.

### Taxonomic implications

Considering that both molecular and chromosomal data consistently recognized well-supported and diagnosable subgroups within the *S. tianschanica* complex, we believe that these groups should be recognized as distinct taxa. The deepest split in the tree is between the N and S clades. The existence of these clades has been demonstrated in several previous studies (Rusin et al., 2018; Cserkész et al., 2019), which were based on limited data sets (*n* = 3–4), however, including two specimens from eastern Tianshan collected in a locality close to terra typica of *S. tianschanica*. In all cases, it was concluded that the distance between the N and S clades corresponded to the inter-specific variation.

Cserkész et al. (2019) described a new species *Sicista zhetysuica* Cserkész, Fülöp, Almerekova, Kondor, Laczkó, Sramkó, 2019 based on two specimens collected near Rudnichny in Dzungar Alatau. The diagnosis rests primarily on the unique sequence o *cytb*, but also includes cytogenetic and morphological information taken from Shenbrot et al. (1995). The comparative genetic data consisted of sequences for a single specimen from Trans-Ili Alatau and two specimens from eastern Tianshan analysed in Pisano et al. (2015), which were all attributed to the nominal form. Although the karyotypes of the type specimens of *S. zhetysuica* were not examined, it was assumed that they belonged to the “Dzungar” karyomorph. The nearest locality with the “Dzungar” karyotype (point #6) is separated from the terra typica of *S. zhetysuica* by a distance of only 20 km. No morphological data on the type specimens of *S. tianschanica* were analysed. Furthermore, relying on the morphometric data from Shenbrot et al. (1995), the authors concluded that *S. zhetysuica* could be distinguished from other Tianshan birch mice by a pronouncedly shorter upper tooth row, which was not the case in our results. The sequences from the cited study were not available in GenBank; nonetheless, it is evident that *S. zhetysuica* corresponds to the N clade, as defined by Lebedev et al. (2019) and in the current study (this follows from the phylogenetic position of the GenBank sequence AF297288 used in both studies). Thus, our molecular results confirm the validity of *S. zhetysuica*.

Furthermore, the species status of the four lineages within the S clade was less evident and needs additional discussion. The *cytb* distances between these lineages (6–8%) fall within the range that may potentially correspond to both inter- and intraspecies divergence in mammals according to commonly accepted criteria (Baker & Bradley, 2006). According to the molecular clock results, the diversification of these lineages dates back to the first part of the Middle Pleistocene. Within *Sicista*, comparable levels of divergence and split ages are found between recognized species pairs, such as *S. strandi*/*S. betulina*, *S. lorigera* (=*S. nordmanni*)/*S. trizona* (Lebedev et al., 2020), *S. klucorica*/*S. caucasica* (Rusin et al., 2018); however, two branches within *S. kluchorica* (with *cytb* distance of 6%) shared similar haplotypes in nuclear genes (Rusin et al., 2018).

Based on the available nuclear data, no signature of gene flow between neighbour karyomorphs (e.g., the “Terskey” and “Talgar”) was detected. Both nuclear genes contained diagnostic substitutions for each of the five genetic lineages. Considering the complex nature of chromosomal differences between these forms (lack of complete chromosome arm homology) one may suppose the existence of a karyotype-mediated reproductive barrier. Taken together, these facts suggest that the “Terskey” and “Talgar” karyomorphs (corresponding to the S2 and S3 lineages) should be ranked as species. Although the karyotype of *S. tianschanica* sensu stricto remains unknown, the high level of genetic differentiation between the nominal form and other lineages is supportive of their species status within the framework of the genetic species concept. To determine the taxonomic position of the data-deficient S4 lineage, additional cytogenetic, morphological, and molecular studies are needed; meanwhile, it seems reasonable to suppose that it may represent a separate, yet undescribed species.

The descriptions of the new taxa follow below. They are regarded as cryptic species because neither of the morphological traits has shown a hiatus between the groups. In particular, no differences in penile morphology, which is essential for species identification in most of *Sicista*, were revealed (Shenbrot et al., 1995). Therefore, the diagnoses rely mostly on chromosomal and molecular characters. The molecular diagnoses are given following the recommendations of Jörger & Schrödl (2013) and Renner (2016). Considering a high level of within-species synonymous variation in mitochondrial genes, diagnostic characters in *cytb* are based on amino acid substitutions.

The *S. tianschanica* species group is considered here to include four species.

#### Sicista tianschanica *(Salensky, 1903)*

Holotype (lectotype): ZIN 2271, skin and skull (def.), male. (Fig. S2).

Paratypes (paralectotypes): ZIN 2272, skin and skull (def.), female; ZIN 2273, skin and skull (def.), female.

The type series was collected in September 1876 by N. M. Przewalski.

The species was described originally as *Sminthus tianschanicus*. The lectotype was designated by Ognev (1948). The name is misspelled as “tianshanica” in some checklists (Holden & Musser, 2005). This should be regarded as “incorrect subsequent spelling”, which is not in prevailing usage (ICZN, Article 33.3).

Terra typica: China, Xinjiang, Ili Prefecture, E Tianshan, Tzanma River valley (“fl. Zanma”).

This locality is listed for ZIN 2271 and ZIN 2272; the locality for ZIN 2273 is indicated as “Tianshan”.

In the original description, Salensky (1903) concluded that *S. tianschanica* is distinguished from other central Asian taxa recognized at that time (*S. concolor* (Büchner, 1892); *S. flavus* (True, 1894); *S. leathemi* (Thomas, 1893) by its larger size. Body size of the holotype is 100 mm (90 and 100 mm in the paratypes). Dorsum is yellowish-brown; venter is grey; chin and throat are nearly white; hindfoot is silver-white; tail is bicolored, darker above and lighter below. Salensky also mentioned several characteristic cranial traits including relatively narrow interparietale and more posteriorly positioned foramina palatina; however, their diagnostic value remain unclear.

Subsequently, Ognev (1948) modified the diagnosis by describing characters of glans penis and baculum that differentiate between *S. tianschanica* and other birch mouse species including externally similar *S. caudata* Thomas, 1907 and *S. napaea* Hollister, 1912. However, this description was based on specimens originating from areas other than East Tianshan; thus, penile morphology of *S. tianschanica* sensu stricto remains unstudied.

According to Ognev (1948) the hindfoot length is 18.7 mm and 19.3 mm in ZIN 2271 and ZIN 2273, respectively. The length of the upper cheek tooth row is 3.73, 3.21 and 3.78 mm for ZIN 2271, 2272 and 2273, respectively. Regarding ZIN 2271 and ZIN 2273, this measurement is larger than in all other examined birch mice of the *S. tianschanica* sensu lato species group.

Distribution: Eastern Tianshan, NE Narat range; exact limits are unknown.

Diagnosis: In this study, we complement the diagnosis of this species with the molecular diagnostic characters for the three examined genes (Tables 2 and 3).

**Table 2:** Molecular diagnostic characters of *Sicista terskeica* sp. nov, *Sicista talgarica* sp. nov. and other taxa of *S. tianschanica* s.l.

	*cytb*, amino acid positions														
	13	16	17	23	39	96	108	209	212	229	230	235	238	246	302
*S. tianschanica*	I	D	S	A	V	L	**I**	**I**	D	T	L	L	I	S	A
*S. zhetysuica*	I	**E**	**A**	A	**I**	M	T	T	N	T	L	**M**	I	S	T
***Sicista*** ***terskeica* sp. nov**	I	D	S	T	L	L	A	T	N	T	**F**	L	T	**F**	A
***Sicista*** ***talgarica* sp. nov.**	M	D	S	T	V	L	T	T	N	**A**	L	L	T	S	A
*Sicista* sp. S5	I	D	S	T	V	L	A	T	N	T	L	L	T	S	T

**Note:**

Numbers correspond to amino acid positions in the *cytb* alignment. Autapomorphies are shown in Bold.

**Table 3:** Molecular diagnostic characters of *Sicista terskeica* sp. nov, *Sicista talgarica* sp. nov. and other taxa of *S. tianschanica* s.l.

	*BRCA1*, nucleotide positions												*IRBP*, nucleotide positions																		
	5	9	40	43	64	66	80	96	156	427	606	621	42	153	227	228	229	267	279	444	618	819	828	831	834	1,035	1,061	1,080	1,082	1,088	1,110
*S. tianschanica*	a	g	a	a	c	t	a	a	c	a	a	t	t	t	t	t	c	t	**a**	**t**	a	t	c	g	t	c	c	c	t	g	c
*S. zhetysuica*	a	**a**	**g**	a	c	**c**	a	**g**	c	a	**g**	**c**	c	**c**	t	c	c	c	c	c	**g**	**c**	**g**	g	**c**	**t**	**t**	**t**	c	**c**	**g**
***Sicista terskeica*** **sp. nov**	a	g	a	**g**	c	t	a	a	c	a	a	t	c	t	t	c	c	t	c	c	a	t	c	g	t	c	c	c	t	g	c
***Sicista talgarica*** **sp. nov.**	**c**	g	a	a	c	t	a	a	c	**g**	a	t	c	t	t	c	c	t	c	c	a	t	c	**a**	t	c	c	c	c	g	c
*Sicista* sp. S4	a	g	a	a	**t**	t	**t**	a	**t**	?	?	?	t	t	**c**	t	**t**	?	?	?	?	t	c	g	t	c	c	c	t	g	c

**Note:**

Numbers correspond to nucleotide positions in reference sequences for *BRCA1* (MK259972) and *IRBP* (KM397153). Autapomorphies are shown in Bold.

#### Sicista zhetysuica *Cserkész, Fülöp, Almerekova, Kondor, Laczkó, Sramkó, 2019*

Type series: the details on the holotype and the paratype are given in Cserkész et al. (2019)

Terra typica: Kazakhstan, Almaty region, Rudnichny village, Dzungar Alatau (Zhetysu Alatau), Koksu Valley,(44°41′N, 78°56′E).

Diagnosis: The karyotype consists of 34 chromosomes (NFa = 54), including five pairs of metacentrics (one large, four small to medium), five pairs of submetacentrics (one small, one large, three medium), one pair of medium-sized subtelocentrics, and five pairs of medium-sized telocentrics. The X-and Y-chromosomes are telocentrics of medium and small-size, respectively. The photograph of chromosomes of a specimen collected ca. 20 km from the type locality (ZMMU S-202212, loc. #5) is given in Sokolov & Kovalskaya (1990; Fig. 1Б). Molecular diagnostic characters are given in Tables 2 and 3.

Distribution: The range consists of two separate parts: the southern (Dzungar Alatau, except the southeast) and the northern (Tarbagatay and Saur).

Description: The length of the upper tooth row is smaller than in *S. tianschanica* sensu stricto (but not smaller than in *S. terskeica* sp. nov. or *S. talgarica* sp. nov.). The width of the palatine bridge is smaller than in *S. terskeica* sp. nov. *S. zhetysuica* differs from *S. terskeica* sp. nov. and *S. talgarica* sp. nov. by a better-developed P4.

White spots on throat and chest are absent (Shenbrot et al., 1995; Cserkész et al., 2019) (Fig. S3).

#### Sicista terskeica sp. nov.

**Figure 7:**
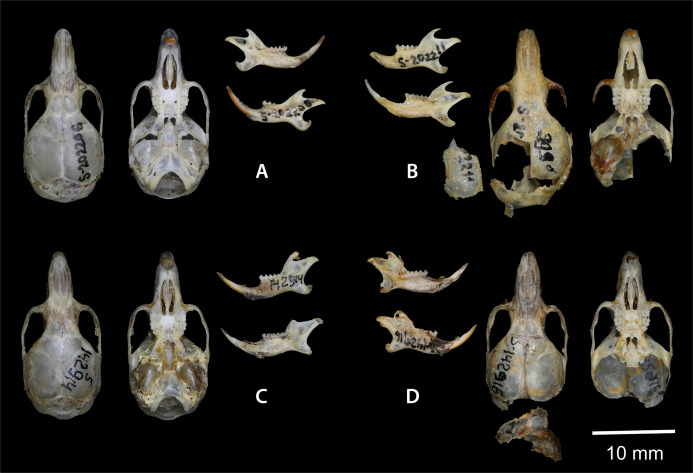
Dorsal and ventral view of the skull, and lingual and labial view of the mandible. (A) the holotype of *Sicista terskeica* sp.nov. (ZMMU S- 202206); (B) the holotype of *Sicista talgarica* sp.nov. (ZMMU S- 202211); (C and D) the paratypes of *S. talgarica* (ZMMU S-142914 and S-142916, respectively). Photos by Vladimir Lebedev & Anastasia Makarova.

Holotype: ZMMU S-202206, skull (Fig. 7A); karyotype photograph published in Sokolov & Kovalskaya (1990; Fig. 1A); male collected on 20 June 1977 by Kovalskaya Yu. M., field number 20–77.

Terra typica: Kyrgyzstan, Issyk-Kul region, Jeti-Oguz (Pokrovka) district, approximately 20 km SW from Jeti-Oguz; Terskey Alatau range, Chon-Kyzyl-Su River valley; approximate latitude and longitude: 42.28 N 78.12 E; altitude 2,700 m.

Holotype measurements: weight 9.8 g, body length 64 mm, tail length 90 mm, hindfoot length 16.5 mm, ear length 15 mm.

Diagnosis: The karyotype consists of 32 chromosomes (NFa = 56). The autosomal complement includes five pairs of metacentrics (one small and three medium to large), six pairs of submetacentrics (one small, one large, four medium), two pairs of medium-sized subtelocentrics, and two pairs of medium-sized telocentrics. The X- and Y-chromosomes are medium- and small-sized telocentrics, respectively. Chromosome morphology is illustrated in Sokolov & Kovalskaya (1990) (Fig. 1A) and Shenbrot et al. (1995) (Fig. 50A). *S. terskeica* differs from *S. zhetysuica* by a different chromosome morphology and different fundamental and chromosome numbers (in the latter 2*n* = 34, NFa = 54). *S. terskeica* differs from *S. talgarica* sp. nov. in the number of metacentric and subtelocentric chromosomes. The species can be distinguished from other species of the *S. tianschanica* species group by the combination of the diagnostic molecular characters as shown in Tables 2 and 3. For the non-introgressed *cytb* haplotypes of *S. terskeica*, the uncorrected distance from the sequence of the holotype (GenBank Acc. No.: MT418278) should not exceed 3.5%.

Etymology: The species is named after the mountain range Terskey Alatau, where the type locality is situated.

Distribution: Northern Tianshan, including Terskey Alatau, Kungey Alatau, Ketmen, and western Trans-Ili Alatau, and central (Inner) Tianshan southward to the Atbashi range.

Description: Cranial, dental, external, and penile characters are as in the other representatives of *S. tianschanica* sensu lato, the description is provided in Vinogradov (1937), Ognev (1948), Shenbrot et al. (1995). According to Shenbrot et al. (1995), body length in adults is 56–70 mm; tail length is 86–103 mm; hindfoot length is 15.4–18.0 mm. Tail length exceeds body length by approximately 50%. Dorsum is drab-brown, tinged with rusty or yellowish to various extent, with no black mid-dorsal stripe. Venter is pale gray; in some specimens, there are white spots on chest and throat. Tail is bicolored, brownish above and whitish below. Upper side of hindfoot is whitish-pale. Glans penis is 4.2–4.6 mm long and ~2.8 mm wide, cylindrical to clavate in shape. Its surface is uniformly covered with small keratin spines; large lateral spines (as in *S. napaea*) or complex additional folds and spikes (as in *S. betulina* or *S. subtilis*) are absent. Baculum is 3.6–3.8 mm long, strongly curved (see Shenbrot et al., 1995; Fig. 49).

Morphometric data suggest that the length of the upper tooth row (2.96–3.30 mm) is smaller than that in *S. tianschanica* sensu stricto. The width of the palatine bridge is greater than in *S. zhetysuica* and *S. talgarica*.

ZooBank registration: urn:lsid:zoobank.org:act:A550FC4E-04ED-4403-ACD2-113F3E95EA67.

#### Sicista talgarica sp. nov.

Holotype: ZMMU S-202211, skull (Fig. 7B); karyotype photograph published in Sokolov & Kovalskaya (1990; Fig. 1Б); male collected in 1987 by Kovalskaya Yu. M., field number 87–55 (999).

**Figure 8:**
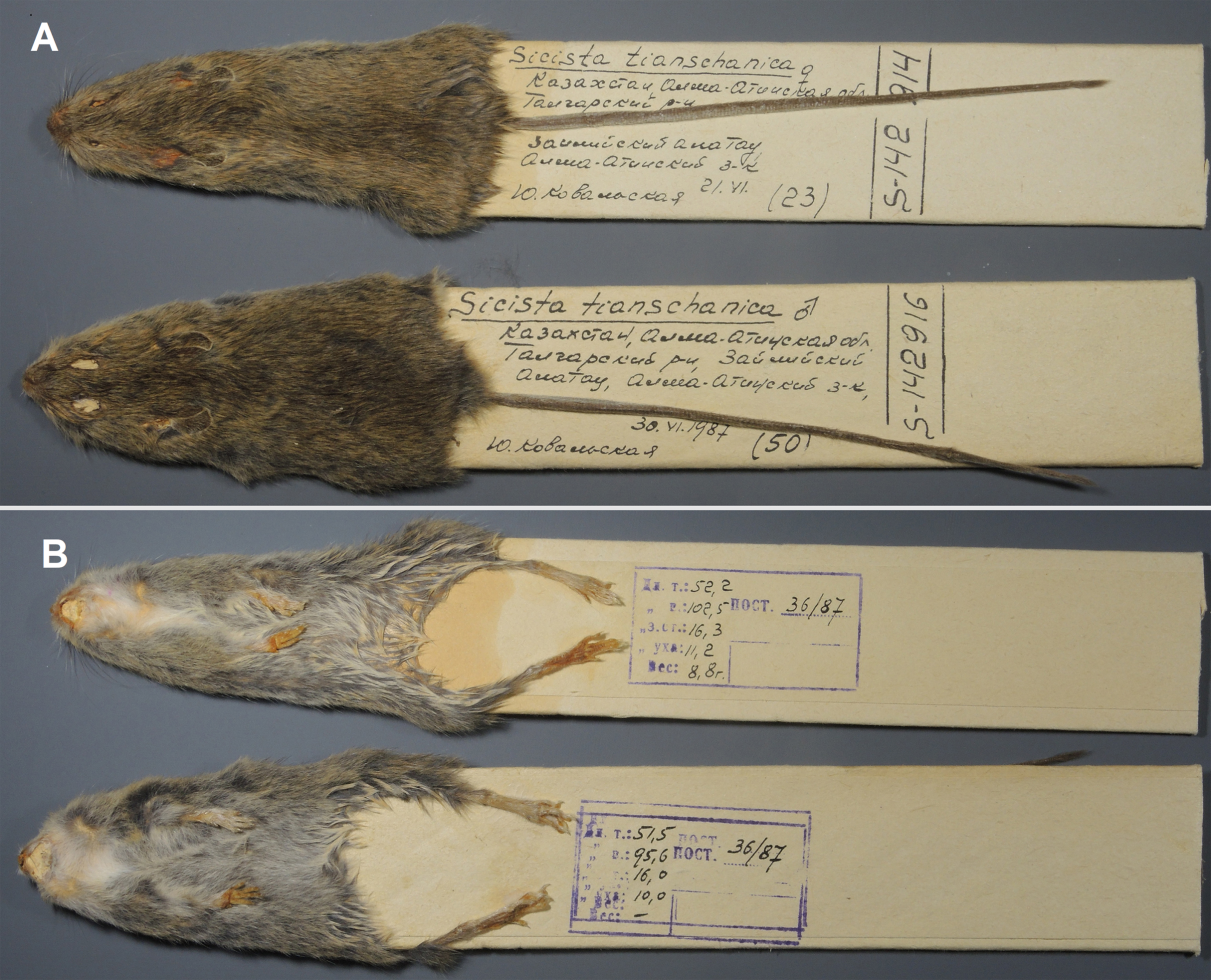
Skins of the paratypes of *S. talgarica* (ZMMU S-142914 and S-142916). Dorsal (A) and ventral (B) view. Photos by Vladimir Lebedev.

Paratypes: ZMMU S-142914, skin and skull (Figs. 8 and 7C), collected 21 June 1987 by Kovalskaya Yu.M., field number 87-23; ZMMU S-142916, skin and skull (Figs. 8 and 7D), collected 30 June 1987 by Kovalskaya Yu. M., field number 87–50.

Terra typica: Kazakhstan, Almaty region, Talgar district, Almaty Natural Reserve, 14 km SE from Talgar; Trans-Ili Alatau, Right Talgar River valley; approximate latitude and longitude: 43.27N, 77.32E; altitude 1,650 m.

Holotype measurements: body length 66 mm, tail length 98 mm, hindfoot length 16 mm, ear length 14.3 mm.

Paratype measurements: S-142914—body length 52.2 mm, tail length 102.5 mm, hindfoot length 16.3 mm, ear length 11.2 mm; S-142916—body length 51.5 mm, tail length 95.6 mm, hindfoot length 16.0 mm, ear length 10.0 mm.

Diagnosis: The karyotype consists of 32 chromosomes (NFa = 56). The autosome set includes six pairs of metacentrics (one large, four medium and one small), six pairs of submetacentrics (one small, four medium and one large), one pair of medium-sized subtelocentrics, and two pairs of medium-sized telocentrics. The X-and Y-chromosomes are telocentrics of medium-and small-sized, respectively. Chromosome morphology is illustrated in Sokolov & Kovalskaya (1990; Figure 1Б). *S. talgarica* differs from *S. zhetysuica* by different chromosome morphology and different fundamental and chromosome number sensu stricto *talgarica* differs from *S. terskeica* sp. nov. in the number of metacentric and subtelocentric chromosomes. Molecular diagnosis is given in Tables 2 and 3. For the non-introgressed *cytb* haplotypes of *S. terskeica*, the uncorrected distance from the sequence of the holotype (GenBank Acc. No.: MT418269) should not exceed 3.5%.

Etymology: The species is named after the type locality.

Distribution: Trans-Ili Alatau–Talgar Mountains and M. Almatinka Valley; eastern limits unknown. The range does not include SE Dzungar Alatau.

Description: Cranial, dental, external, and penile characters match with that of *S. terskeica* sp. nov., as described above. According to Shenbrot et al. (1995), body length in adults is 57–73 mm; tail length is 102–113 mm (appr. 160% of body length); hindfoot length is 17.0–19.0 mm. Animals with white spots on throat and chest are more common in *S. talgarica* than in *S. terskeica* sp. nov. (Fig. 7; Fig. S3) .

The length of the upper tooth row (3.04–3.32 mm) is shorter than that in *S. tianschanica* sensu stricto. The width of the palatine bridge is smaller than that in *S. terskeica* sp. nov.

ZooBank registration: urn:lsid:zoobank.org:act:0C088A48-49D6-4CA9-BD1A-C87B9DA46808.

## Conclusions

The present work is the first study to examine genetic variation in a small mammal species in the Tianshan region, the mammal fauna of which, as well as of other Central Asian mountains, is still known insufficiently. The fact that this area harbours two Pliocene-age lineages of *S. tianschanica* sensu lato highlights its role as a long-term refuge and a potential centre of diversification. The progress of future taxonomic research on hard to reach Central Asian species (e.g., *Sicista concolor* from NE Tibet and Kashmir) critically depends on the availability of historical DNA samples of museum specimens including types.

## Supplemental Information

10.7717/peerj.10759/supp-1 Supplemental Information 1 Alignments of irbp gene.

10.7717/peerj.10759/supp-2 Supplemental Information 2 Alignments of brca1 gene.

10.7717/peerj.10759/supp-3 Supplemental Information 3 Alignments of cytb gene.

10.7717/peerj.10759/supp-4 Supplemental Information 4 GenBank accession numbers for the original sequences generated in this study.

10.7717/peerj.10759/supp-5 Supplemental Information 5 Tables S1–S4 and Figs. S1–S3.

## Additional Information and Declarations

### DNA Deposition

The following information was supplied regarding the deposition of DNA sequences: Raw data are available at GenBank: MT418257–MT418314.

### Data Availability

The following information was supplied regarding data availability: Alignments of cytb, IRBP and BRCA1 are available in the Supplemental Files.

### New Species Registration

The following information was supplied regarding the registration of a newly described species: Publication LSID: urn:lsid:zoobank.org:pub:6F34EAF8-92B3-42BC-B07E-E6C16731A9A1. *Sicista terskeica* sp. nov. LSID: urn:lsid:zoobank.org:act:A550FC4E-04ED-4403-ACD2-113F3E95EA67. *Sicista talgarica* sp. nov. LSID: urn:lsid:zoobank.org:act:0C088A48-49D6-4CA9-BD1A-C87B9DA46808.
