# Supplementary material for: Molecular systematics of the Sicista tianschanica species complex: a contribution from historical DNA analysis
Source: PeerJ. 2021 Jan 12;9:e10759. doi: 10.7717/peerj.10759 (PMC7810041; doi:10.7717/peerj.10759)

SUPPLEMENTARY MATERIALS

Article “Molecular systematics of the *Sicista tianschanica* species complex: A contribution from historical DNA analysis”

Lebedev et al., 2020.

Table S1:

Primers used in the study.

Gene	Primer name	Sequence (5`-3`)	Source
cytb	L233	TAATYCGCTATCTCCACGCCAA	this study
	L400	CCATGAGGMCAAATRTCATTCTGAGG	
	L471	CACCACCCTYGTYGAATGAATCTGA	
	L516_S	AGCCACCTTAACACGATTCTTC	
	L754	GACCCAGACAACTATATACCTGCCAACC	
	L754_S	GACCCYGACAACTAYATACCTGCCAAC	
	L7	ACCAATGACATGAAAAATCATCGTT	Montgelard et al., 2002
	L64	CCAGCYCCCTCCAAYATCTCCTC	this study
	L862_N	GCGTAATTGCCCTCGTACTCTCYATC	
	L800_S	CATCAAACCAGAGTGGTACTTCCTATT	
	L974	TCTGARTCCTAGTTGCTGACCTAT	
	L654	CCCMTTYCACCCCTACTACACCAT	
	H575b	RTGGAGGAAAATTARGTGTACTATGGC	
	H484	TCTACGGAGAAGCCGCCTCAGATTCATTC	
	H760	GGGTTGGCAGGTATRTAGTTGTC	
	H929	GCGGAATGTTATACTTCGTTGTTTTG	
	H281_N	CCTCGGCCAACGTGCATAAATAG	
	H271_S	CCCACGTGAAGRAATAAGCAGATGAA	
	H161	GGCTGTCAGGGTRTCAGATGTGTAGT	
	H6	TCTCCATTTCTGGTTTACAAGAC	Montgelard et al., 2002
	H1042	TMGATGCGATTTGTCCRATGATAA	this study
	H927	GCGGAAKGTTATRCTTCGTTGTTTTGAG	
BRCA1	F90b	TGAAAGTAAGGAAACATGTAATGAGAGAC	
	R220a	CCAAGCAAGCTCTTCATTATCTCT	
	F127a	CAGCAATGAGAAGAAGGCGGAACT	
	R350a	CATTTGAAACGTCTACGGCACCA	
	F285a	GGTTTTCCAGAAGTGGTGAAATGTTA	
	R475a	CCCAAATATTTTATCTTTGATATTAGTCT	
	F455a	AGTGAAAGAGTCTGCTCCAAACCA	
	R630a	AGGCCTGATATACCTCTCCTTTTAC	
	F174b	GGGTTTCKGTTCATTCGATTCTTGTCTC	
	R100	TGTGGCACAGATGTTCRTGCCAGCTCATTACA	Lebedev et al., 2013
IRBP	F25	GGCCATCCARCAGGTAATGAAGAG	Lebedev et al., 2013
	F34	CCATCCAGCAGGYCATGAAGAGTC	this study
	R229	TAGATAACCCACATTGCCCTCCAG	
	F205	CTGGTCCAGCTGCAGAAGAACAT	
	R448	GGARGGGCGATTGTAGATGGTGTC	
	F400	GTATCCCCTATGTCATCTCCTACTTGC	
	R694	CTGGACACAGGCACYGTGAGGAA	
	F597	GGAYATCACCTACATCCTCAAACAGA	
	R878	ACGGTCCACCAGTGTGTAGTAATCCT	
	F730	CTGGGCGGAGGCGGACAAACAT	
	R946	RAGATCCTCCTCGGAGACCACAGC	
	F867	GGAGGCCCTTCAGGATTACTACACACT	
	R1175	CACTGACACYTCAAACACAGAGTCCAC	
	F923	ACCACCTGGCCAGCATGGACTACT	
	R1180	CCTGGTAGCACTGACACYTCAAACACA	

Table S2:
Primer combinations used for amplification of fragments of *cytb*, *IRBP*, and *BRCA1*.

Gene	Fragment	Primer combinations	Size (bp)	First position	Last position	Annealing temperature
cytb	1	L7 / H161	160	<1	160	54
	2 North	L64 / H281_N	193	87	279	55
	2 South	L64 / H271_S	184	87	270	55
	3	L233 / H484	229	255	483	55
	4	L400 / H575b	151	426	576	54
	5 North	L471 / H760	264	496	759	55
	5 South	L516_S / H760	222	538	759	54
	6A	L654 / H927	249	678	926	56
	6 North	L754 / H929	147	782	928	54
	6 South	L754_S / H929	148	781	928	56
	7 North	L862_N / H1042	148	895	1042	52
	7 South	L800_S / H1042	215	828	1042	55
	8	L974 / H6	143	998	>1140	52
	1.2	L64 / H161	74	87	160	51
	4.5	L471 / H575b	81	496	576	53
	5.5	L654 / H760	83	678	759	51
	6.7	L800_S / H929	101	828	928	52
BRCA1	1A	F90b / R220a	98	146	243	54
	1B	F100 / R220a	243	1	243	55
	1C	F100 / R174	179	1	179	55
	2	F127a / R350a	204	177	380	56
	3	F285a / R475a	170	331	500	52
	4	F455a / R630a	154	499	652	51
IRBP	1A	F25 / R229	202	39	240	59
	1B	F34 / R229	200	41	240	59
	2	F205 / R448	226	228	453	59
	3	F400 / R694	266	428	693	59
	4	F597 / R878	267	611	877	60
	5	F730 / R946	194	752	945	62
	6A	F867 / R1175	241	894	1134	61
	6B	F923 / R1180	199	944	1142	61

Table S3:
Substitution employed in the ML and Bayesian analyses of the cytb data.

codon positions	1st	2nd	3rd
ML in IQTREE	K3P+I	HKY	TIM3
BI in MrBayes	GTR+I	HKY	GTR

Table S4:
Cranial measurements of *Sicista tianschanica* s.l. and results of the significance tests for between-group differences (original P-values / Bonferroni adjusted P-values). Values significant at P<0.05 after Bonferroni correction are shown in bold.

"Dzungar" n=17	"Terskey" n=20	"Talgar" n=14	"Dzungar" vs. "Terskey"	"Dzungar" vs. "Talgar"	"Talgar" vs. "Terskey"	Effect of group (GLM with CIL effect removed)
mean ± standard deviation (min – max)			Mann-Whitney U-test			F-test
18.46 ± 0.501 (17.41 - 18.98)	18.85 ± 0.691 (16.76 - 20.10)	18.79 ± 0.596 (17.97 – 20.00)	P<0.05 / ns	ns	ns	–
6.16 ± 0.157 (5.87 - 6.55)	6.41 ± 0.232 (5.90 - 6.73)	6.40 ± 0.246 (6.12 - 7.03)	**P<0.001 / P<0.05**	P<0.01 / ns	ns	P<0.01 / ns
4.24 ± 0.161 (3.92 - 4.52)	4.34 ± 0.189 (3.94 - 4.64)	4.40 ± 0.136 (4.17 - 4.62)	ns	P<0.01 / ns	ns	ns
3.22 ± 0.148 (2.94 - 3.46)	3.4 ± 0.147 (3.09 - 3.66)	3.21 ± 0.132 (2.96 - 3.39)	**P<0.001 / P<0.05**	ns	**P<0.01 / P<0.05**	**P<0.001 / P<0.01**
6.61 ± 0.216 (6.23 - 6.93)	6.78 ± 0.366 (5.77 - 7.25)	6.87 ± 0.335 (6.43 - 7.56)	ns	P<0.05 / ns	ns	ns
3.87 ± 0.119 (3.72 - 4.09)	4.00 ± 0.102 (3.79 - 4.17)	3.89 ± 0.125 (3.72 - 4.12)	**P<0.01 / P<0.05**	ns	P<0.05 / ns	P<0.01 / ns
7.04 ± 0.253 (6.45 - 7.33)	7.05 ± 0.232 (6.73 - 7.71)	7.08 ± 0.222 (6.75 - 7.48)	ns	ns	ns	ns
1.96 ± 0.057 (1.82 - 2.08)	1.96 ± 0.067 (1.82 - 2.12)	2.00 ± 0.047 (1.92 - 2.08)	ns	P<0.05 / ns	ns	ns
2.37 ± 0.081 (2.14 - 2.46)	2.37 ± 0.100 (2.22 - 2.59)	2.50 ± 0.075 (2.40 - 2.70)	ns	**P<0.001 / P<0.001**	**P<0.001 / P<0.01**	**P<0.001 / P<0.001**
3.74 ± 0.069 (3.61 - 3.83)	3.63 ± 0.104 (3.45 - 3.86)	3.84 ± 0.139 (3.63 - 4.03)	**P<0.01 / P<0.05**	P<0.05 / ns	**P<0.001 / P<0.01**	**P<0.001 / P<0.001**
3.77 ± 0.076 (3.61 - 3.92)	3.81 ± 0.144 (3.52 - 4.03)	3.75 ± 0.142 (3.43 - 4.06)	ns	ns	ns	ns
2.18 ± 0.052 (2.12 - 2.29)	2.36 ± 0.106 (2.12 - 2.56)	2.21 ± 0.094 (2.03 - 2.41)	**P<0.001 / P<0.001**	ns	**P<0.001 / P<0.01**	**P<0.001 / P<0.001**
1.44 ± 0.066 (1.34 - 1.54)	1.37 ± 0.067 (1.26 - 1.51)	1.46 ± 0.08 (1.31 - 1.56)	P<0.01 / ns	ns	P<0.01 / ns	**P<0.001 / P<0.001**
3.10 ± 0.098 (2.92 - 3.26)	3.13 ± 0.101 (2.96 - 3.30)	3.18 ± 0.085 (3.04 - 3.32)	ns	P<0.05 / ns	ns	ns
2.61 ± 0.084 (2.45 - 2.78)	2.69 ± 0.082 (2.58 - 2.86)	2.75 ± 0.07 (2.61 - 2.85)	P<0.05 / ns	**P<0.001 / P<0.01**	P<0.05 / ns	**P<0.001 / P<0.01**
1.05 ± 0.039 (0.99 - 1.11)	1.03 ± 0.043 (0.97 - 1.14)	1.11 ± 0.041 (1.02 - 1.19)	ns	P<0.05 / ns	ns	**P<0.001 / P<0.001**
0.63 ± 0.043 (0.53 - 0.72)	0.72 ± 0.038 (0.66 - 0.80)	0.67 ± 0.036 (0.62 - 0.73)	**P<0.001 / P<0.001**	P<0.01 / ns	**P<0.001 / P<0.05**	**P<0.001 / P<0.001**
1.00 ± 0.029 (0.94 - 1.05)	1.01 ± 0.038 (0.97 - 1.09)	1.05 ± 0.039 (0.98 - 1.12)	ns	**P<0.01 / P<0.05**	P<0.05 / ns	P<0.01 / ns

0.72 ± 0.038 (0.64 - 0.78)	0.77 ± 0.041 (0.70 - 0.84)	0.75 ± 0.023 (0.72 - 0.79)	**P<0.001 / P<0.05**	P<0.05 / ns	ns	**P<0.01 / P<0.05**
3.00 ± 0.107 (2.79 - 3.19)	3.09 ± 0.093 (2.87 - 3.26)	3.15 ± 0.083 (3.04 - 3.31)	P<0.05 / ns	**P<0.001 / P<0.01**	P<0.05 / ns	**P<0.01 / P<0.05**
1.14 ± 0.035 (1.09 - 1.21)	1.16 ± 0.05 (1.02 - 1.26)	1.19 ± 0.029 (1.14 - 1.23)	ns	**P<0.001 / P<0.05**	P<0.05 / ns	P<0.05 / ns
0.84 ± 0.056 (0.74 - 0.95)	0.91 ± 0.03 (0.85 - 0.97)	0.87 ± 0.029 (0.82 - 0.92)	**P<0.001 / P<0.05**	P<0.05 / ns	P<0.01 / ns	**P<0.001 / P<0.05**
0.88 ± 0.027 (0.83 - 0.92)	0.86 ± 0.034 (0.80 - 0.92)	0.91 ± 0.035 (0.86 - 0.98)	ns	P<0.01 / ns	**P<0.001 / P<0.01**	**P<0.001 / P<0.001**
0.91 ± 0.028 (0.86 - 0.95)	0.91 ± 0.047 (0.82 - 0.98)	0.93 ± 0.031 (0.88 - 0.99)	ns	P<0.05 / ns	ns	ns
1.19 ± 0.013 (1.16 - 1.21)	1.16 ± 0.01 (1.14 - 1.18)	1.16 ± 0.008 (1.14 - 1.17)	**P<0.001 / P<0.001**	**P<0.001 / P<0.001**	ns	–

List of cranial measurements

1. condylo-incisive length (CIL)
2. zygomatic length (ZL)
3. incisive foramen length (IFL)
4. palatine bridge length (PBL)
5. postpalatine length (PPL)
6. palatine bridge width (PBW)
7. zygomatic width at infraorbital foramena (ZFW)
8. foramen magnum width (FMW)
9. mesopterygoid fossa width (MEPFW)
10. incisive width (IW)
11. nasal width (NASW)
12. interorbital width (IOW)
13. auditory bulla width (BULW)
14. maxillary toothrow length (P4M3L)
15. maxillary molar row length (M1M3L)
16. upper M1 length (M1L)
17. upper M3 length (M3L)
18. upper M2 width (M2W)
19. upper M3 width (M3W)
20. mandibular molar row length (LM1M3L)
21. lower M2 length (LM2L)
22. lower M3 length (LM3L)
23. lower M1 width (LM1W)
24. lower M2 width (LM2W)

List of specimens used in the morphometric analysis (ZMMU collection). Numbers shown in bold belong to specimens included in the molecular analyses. Underlined collection numbers denote animals with known karyotype. Underlined geographic names correspond to karyotyped localities.

"Dzungar"
Kazakhstan:
Dzungar Alatau, Koktal riv. S-146654; Dzungar Alatau, Lepsy (Lepsinsk) S-96480, S-96505, S-96506; Tarbagatai, Urzhar dis. S-146635, S-146636, S-146638, S-146640, S-146641, S-146642, S-146644, S-146645, S-146646, **S-146647**, S-146648; Saur, Zhanturmys **S-146650**, S-146652;
"Terskey"
Kazakhstan:
Ketmen, Syumbe **S-148430**; Terskey Alatau, Raiymbek (Narynkol) dis. S-149918; Terskey Alatau, Bayankol riv. **S-148432**, **S-148433**, S-149917;
Kyrgyzstan:
Central Tianshan, Kara-Kiche **S-148425**; Central Tianshan, Akbeit can. S-58777, S-58785; Issyk-Kul reg., Tyup riv. S-136506, S-136508, S-136509, S-136512; Terskey Alatau - Saryzhaz, M. Taldysu riv. **S-148428**, S-148434, S-149919; Terskey Alatau, Chon-Kyzyl-Su S-49403, **S-202206**; Terskey Alatau, Karkara bas., Uchkashka riv. **S-148426**, S-148427, **S-148429**;
"Talgar"
Kazakhstan:
Trans-Ili Alatau, Talgar dis., Almaty nat.res. **S-142913**, **S-142914**, S-142915, S-145668; Trans-Ili Alatau, M Almaatinka riv. **S-54768**; Trans-Ili Alatau, S of Almaty S-26149, S-90421, S-90423, S-90424, S-90426, S-90427, S-90433, S-90434, S-90436;

Supplementary Figure S1.
Schematic representations of karyotypes reported for *Sicista tianschanica* sensu lato. The schemes were drawn based on images from Shenbrot et al. (1995), Figure 50.
Abbreviations: M - metacentric, SM – submetacentric, ST – subtelocentric, A –acrocentric.

The three karyomorphs are distributed allopatrically; no interpopulation variation has been revealed. The "Dzungar" karyotype (C) is highly differentiated from the other two in chromosome structure, it has a different number of autosomal arms (NFa=54), thus demonstrating that the variation among karyotypes could not be produced by Robertsonian rearrangements (whole arm fusions/fissions) only. The "Terskey" and "Talgar" karyotypes share the same NFa=56; however, they differ in the number of metacentric and subtelocentric chromosomes. Homology of chromosome arms can not be established for at least one chromosome pair (supposedly the one marked with an asterisk), which indicates that these two karyomorphs, differ by a non-Robertsonian rearrangement. To identify synteny blocks and reconstruct the sequence of evolutionary chromosomal changes additional studies using differential staining or chromosome painting are necessary.
The karyotypic sample includes 64 specimens from 15 localities, local samples consist of 1 to 15 specimens (Sokolov and Kovalskaya, 1990; Shenbrot et al., 1995; Kovalskaya, unpublished data).
"Dzungar" karyomorph:
Dzungar Alatau - 4 localities, 11 specimens;
Tarbagatay – 1 locality, 6 specimens;
Saur - 1 locality, 4 specimens.
"Terskey" karyomorph:
N and C Tianshan – 7 localities, 33 specimens.
"Talgar" karyomorph:
Trans-Ili Alatau - 1 locality, 9 specimens;
Dzungar Alatau - 1 locality, 1 specimen.

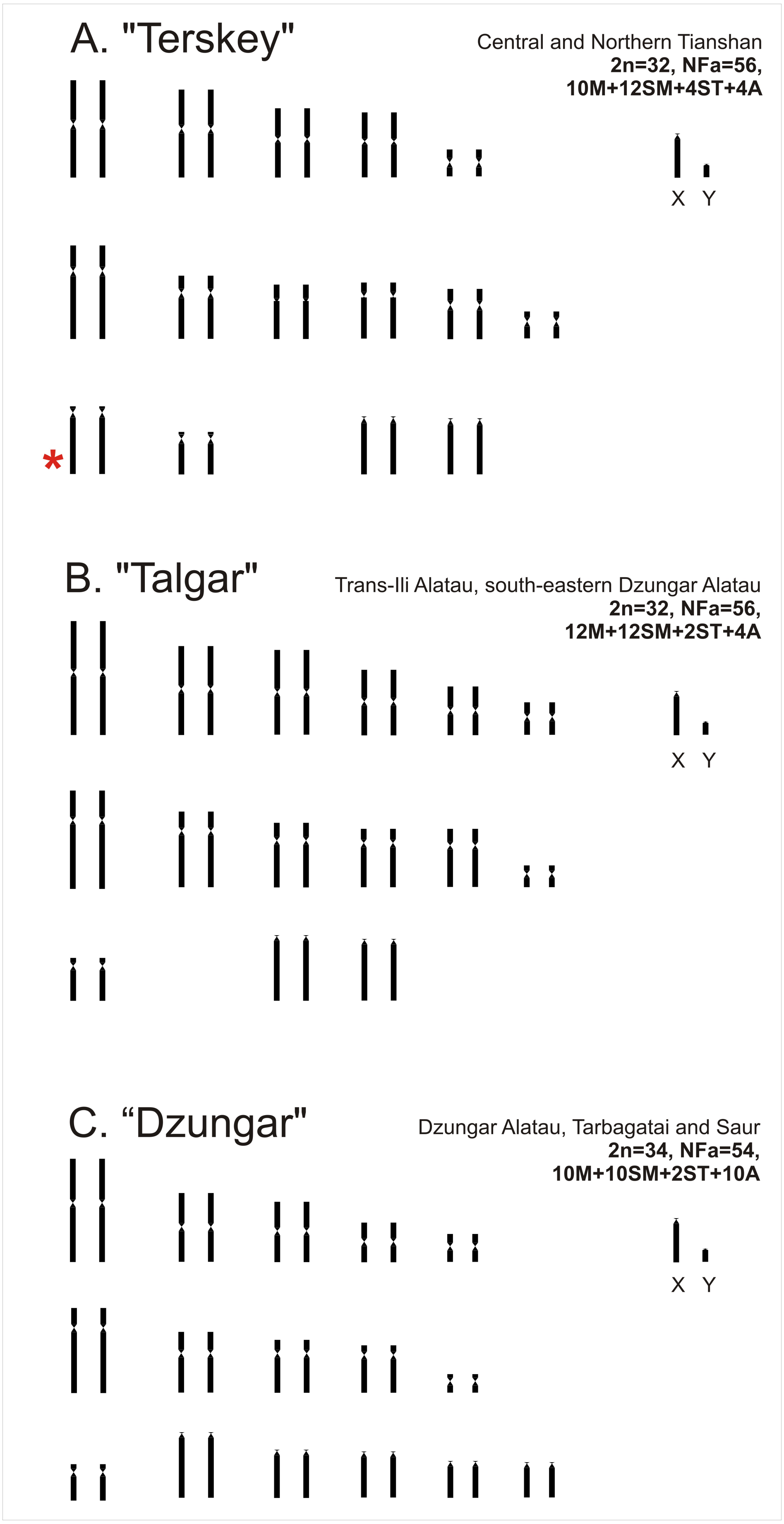
A. "Terskey"
Central and Northern Tianshan
2n=32, NFa=56,
10M+12SM+4ST+4A
X Y
*
B. "Talgar"
Trans-Ili Alatau, south-eastern Dzungar Alatau
2n=32, NFa=56,
12M+12SM+2ST+4A
X Y
C. “Dzungar"
Dzungar Alatau, Tarbagatai and Saur
2n=34, NFa=54,
10M+10SM+2ST+10A
X Y

Supplementary Figure S2.

Dorsal and ventral view of the skull and skin of the type of *Sicista tianschanica* Salensky,1903 (ZIN 2271). Photos by Vladimir Lebedev.

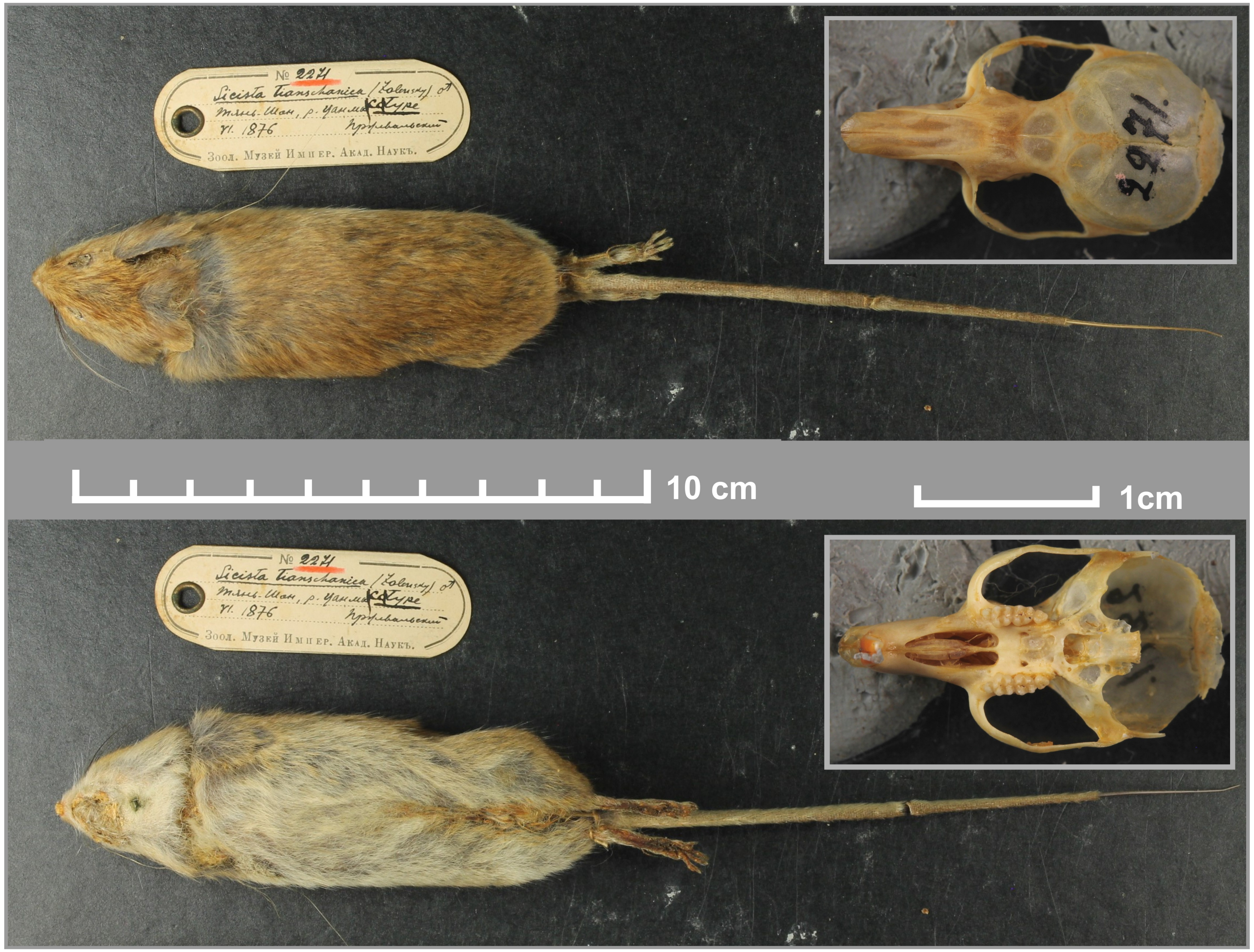

Supplementary Figure S3.
Above: Skins of specimens of *S. terskeica* sp. nov. (ZMMU S-146428 and S-148432). These specimens are not included in the type series of *S. terskeica*.
Below: Skins of specimens of *S. zhetysuica* (ZMMU S-146647 and S-146655). Photos by Vladimir Lebedev.

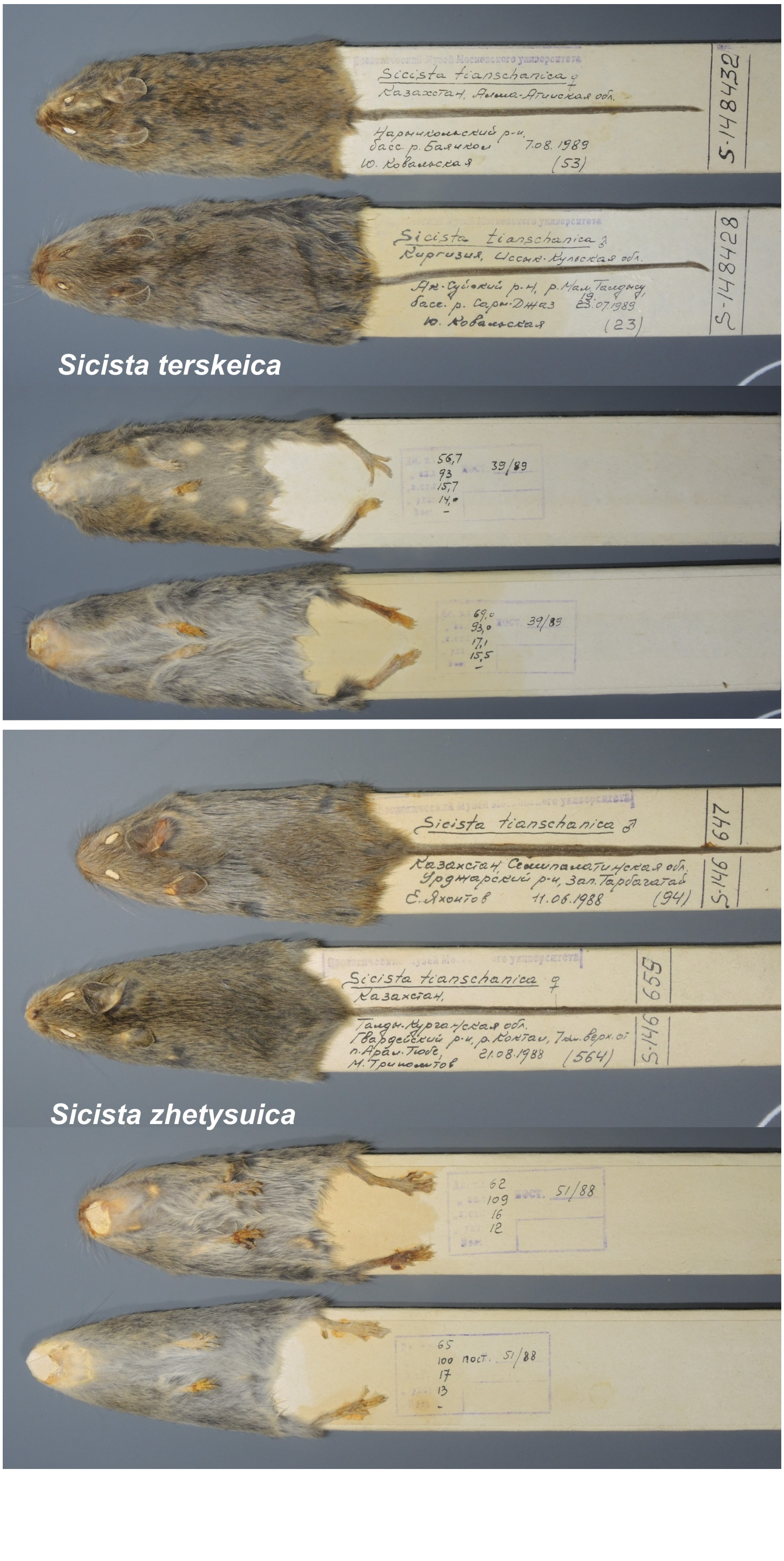

Supplement: Supplemental Information 5 [file peerj-09-10759-s005.pdf]
